# Efficacy and safety of pharmacological interventions in second- or later-line treatment of patients with advanced soft tissue sarcoma: a systematic review

**DOI:** 10.1186/1471-2407-13-385

**Published:** 2013-08-13

**Authors:** Sheetal Sharma, Shweta Takyar, Stephanie C Manson, Sarah Powell, Nicolas Penel

**Affiliations:** 1 Heron Health Pvt. Ltd., Chandigarh, India; 2 GlaxoSmithKline, Uxbridge, UK; 3 Department of General Oncology, Centre Oscar Lambret, Lille, France

**Keywords:** Systematic review, Pazopanib, Soft tissue sarcoma

## Abstract

**Background:**

Current guidelines recommend anthracycline-based chemotherapy primarily with doxorubicin either as monotherapy or in combination with ifosfamide as the first-line treatment for most advanced STS subtypes. Therapeutic options after failure of doxorubicin and/or ifosfamide are limited. This study aimed to comprehensively review available data on the activity and safety of interventions in second- or later-line treatment of advanced STS.

**Methods:**

Electronic literature databases (Embase^®^, MEDLINE^®^, MEDLINE^®^ In-Process, Cochrane Central Register of Controlled Trials, and Cochrane Database of Systematic Reviews) were searched from 1980 to 01 March 2012 to identify randomised controlled trials (RCTs) and non-randomised studies (both prospective and retrospective) evaluating pharmacological interventions in patients with advanced STS pre-treated with anthracycline- and/or ifosfamide-based therapy.

**Results:**

The review identified six RCTs (one phase III and five phase II trials) and 94 non-randomised studies. Based on the primary trial endpoints, RCTs demonstrated favourable efficacy for pazopanib over placebo (PFS: 4.6 months vs. 1.6 months), gemcitabine plus dacarbazine over dacarbazine monotherapy (3-month PFS rate: 54.2% vs. 35.2%), and trabectedin 3-weekly schedule over weekly schedule (TTP: 3.7 months vs. 2.3 months. The non-randomised studies demonstrated heterogeneity in efficacy and safety results.

**Conclusions:**

Across the RCTs, pazopanib over placebo, gemcitabine-dacarbazine over dacarbazine, and trabectedin 3-weekly over weekly regimen clearly demonstrated a PFS advantage in the second- and later-line treatment of advanced STS. With only one phase III trial in this setting, there is a clear need for additional comparative trials to better understand the risk: benefit ratios of available agents and combinations.

## Background

Soft tissue sarcomas (STS) are a heterogeneous group of rare tumours that arise predominantly from the embryonic mesoderm [1]. STS has more than 50 distinct histological subtypes, with leiomyosarcoma, liposarcoma, synovial sarcoma, undifferentiated pleomorphic sarcoma, and malignant peripheral nerve sheath tumours being among the most common subtypes [1]. STS occurs rarely and accounts for approximately 1% of malignancies in adults and 2% of cancer mortality [2,3]. Nearly half of the patients diagnosed with STS develop advanced/metastatic disease and eventually die from the disease [4]. Patients typically demonstrate a median survival ranging from 11 to 18 months from diagnosis of advanced disease [5,6].

The treatment for STS is largely dictated by the tumour grade, size, location of metastatic sites, and the histological subtype [4,6,7]. Outside of clinical trials, cytotoxic chemotherapy is the only available systemic therapy for patients with advanced disease and its goal is primarily palliative [6]. Current guidelines including the European Society for Medical Oncology and the National Comprehensive Cancer Network treatment guidelines recommend anthracycline-based chemotherapy - primarily with doxorubicin, either as monotherapy or in combination with ifosfamide, as the first-line treatment for most advanced STS subtypes [7,8]. Therapeutic options after failure of doxorubicin and/or ifosfamide are limited and there are no standard recognised therapies. Options used in clinical practice include ifosfamide, trabectedin, gemcitabine in combination with docetaxel, and dacarbazine-based regimens [9]. With the advent of new targeted therapies for treatment of advanced STS, it is important to understand the current evidence base in this setting. We aimed to comprehensively review available data on the efficacy and safety of treatments used for patients with advanced STS pre-treated with anthracycline- and/or ifosfamide-based therapy. The comparability amongst this evidence was examined in light of recent Phase III trial evidence for pazopanib, a new oral selective tyrosine kinase inhibitor for the treatment of advanced STS.

## Methods

In order to provide a robust assessment of the available evidence, a systematic review was undertaken to identify, describe and interpret the current state of evidence. The review was conducted in accordance with the Preferred Reporting Items for Systematic Reviews and Meta-Analyses (PRISMA) guidelines (See Additional file 1) [10].

### Searching

The review was based on a comprehensive search of MEDLINE^®^, including MEDLINE^®^ In-Process, Embase^®^, Cochrane Central Register of Controlled Trials (CENTRAL), and Cochrane Database of Systematic Reviews from 1980 to 01 March 2012. An additional file describes the search strategy used for MEDLINE^®^ and Embase^®^ (see Additional file 2).

In addition to the literature database search, abstracts from conference proceedings including American Society of Clinical Oncology, European Society for Medical Oncology, European Conference for Clinical Oncology, Connective Tissue Oncology Society and Musculoskeletal Tumour Society were hand searched from 2007 to March 2012. For trials in progress, Clinicaltrials.gov, UK clinical trials gateway, and International Standard Randomised Controlled Trial Number were searched. Bibliographic searching of included trials and systematic reviews was also performed.

### Study selection and characteristics

The review included randomised controlled trials (RCTs) and non-randomised studies (prospective and retrospective studies) in patients with pre-treated advanced STS. The review was limited to studies in which patients had received prior anthracycline and/or ifosfamide therapy since these are generally considered to be the standard of care for the first-line treatment of advanced STS [7-9]. References were excluded from the review if they recruited paediatric patients (<18 years old). Studies exclusively enrolling patients with gastrointestinal stromal tumours (GIST), Kaposi sarcoma, and Ewing’s family of tumours were also excluded because of their unique biology and management compared with other STS subtypes. Studies that recruited a mixed STS population including GIST, Kaposi sarcoma, or Ewing tumours with no appropriate subgroup data by histological subtype were excluded. Other exclusion criteria were where no subgroup data for patients with advanced stage STS were available across trials recruiting both patients with early stage and advanced STS, or where studies included a mixed population of treatment-naive patients and patients previously treated with anthracycline- and/or ifosfamide-based therapy with no subgroup data for the pre-treated patients.

Further, to be included in the review, studies were required to be published in English and investigating a therapy identified either in STS treatment guidelines [6-8,11], cited in STS treatment review papers [2,12,13] or being researched in the pre-treated advanced STS setting (carboplatin, cyclophosphamide, dacarbazine, docetaxel, doxorubicin, epirubicin, etoposide, gemcitabine, ifosfamide, liposomal doxorubicin, paclitaxel, pazopanib, trabectedin, vincristine, cisplatin, vinblastine, methotrexate, tamoxifen, sunitinib, sorafenib, deforolimus, temsirolimus, everolimus, gefitinib, erlotinib, cetuximab, or brostallicin alone or in combination). Comparative studies were included if the intervention of interest was compared with placebo, best supportive care, or any of the included interventions.

All studies retrieved by searches were screened according to above defined eligibility criteria. Initial screening of the retrieved citations was conducted independently by two reviewers on the basis of the title and abstract. Any discrepancies between the reviewers were resolved by a third independent reviewer. The full-text publications of all citations of potential interest were then screened for inclusion by two reviewers (SS and ST), with disagreements resolved by a third independent reviewer.

### Validity assessment

Quality assessment of RCTs was performed using a comprehensive critical appraisal tool based on the National Institute for Health and Clinical Excellence’s and Cochrane’s critical appraisal tool [14,15]. Critical appraisal of comparative studies (other than RCTs) and single-arm studies was conducted using the Downs and Black checklist [16]. The data endpoints extracted included overall survival (OS), progression-free survival (PFS), overall response rate (ORR), complete response (CR), partial response (PR), stable disease (SD), progressive disease (PD), time to progression (TTP), duration of response (DOR), time to response (TTR), EORTC Quality of Life-Questionnaire-C30 score, EQ-5D score, adverse events, and withdrawals.

### Data abstraction

Relevant data from all included studies were extracted using a pre-defined extraction grid. Data extraction was conducted in parallel by two independent reviewers with any differences resolved by a third independent reviewer. Where more than one publication was identified that described a single trial, the data were compiled into a single entry to avoid double counting of patients.

### Quantitative data synthesis

There was considerable heterogeneity across studies in terms of interventions, comparisons, patient population, and study designs. Further, the available evidence base was limited with no more than one study directly comparing the same set of interventions. Therefore meta-analysis, indirect, and mixed treatment comparison of the included interventions were not appropriate. We describe the results qualitatively with detailed results presented in supporting tables. The results are presented separately for RCTs and non-randomised studies.

## Results

### Trial flow / flow of included studies

**Figure 1:**
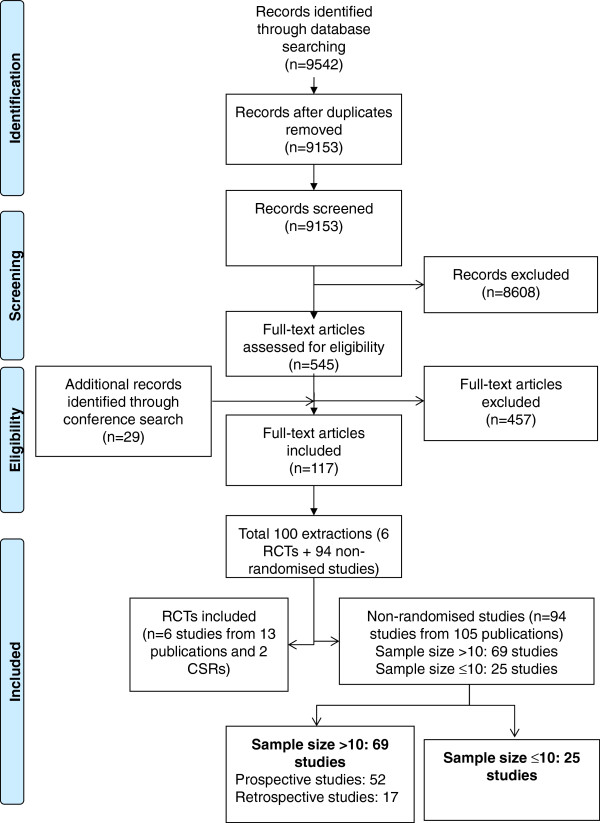
**Flow of studies through the systematic review process.** The figure describes the flow of studies through the review, according to PRISMA guidelines. The search of the literature databases yielded 9542 separate references. Following the screening of abstracts and full-text publications against the inclusion/exclusion criteria a total of six RCTs (reported in 13 publications) and 94 non-randomised studies (reported in 105 publications) met the inclusion criteria for the review.

The flow of studies through the review, according to PRISMA guidelines [17], is shown in Figure 1.

The search of the literature databases yielded 9542 separate references. Following the screening of abstracts against the inclusion/exclusion criteria, 545 full-text reports were obtained for detailed evaluation. Additionally, 29 references meeting the inclusion/exclusion criteria for the review were identified from conference proceedings. After screening, a total of six RCTs (reported in 13 publications and two Clinical Study Reports) and 94 non-randomised studies (reported in 105 publications) met the inclusion criteria for the review. The list of the 457 studies excluded from the review along with exclusion rationale is available on request.

### RCTs

#### Study characteristics

The key patient and study design characteristics of the six included RCTs are presented in Table 1. All included RCTs were Phase II trials, except for the PALETTE study, which was the only Phase III RCT [18,19]. All the studies included in the review aimed to evaluate the activity and safety of the interventions under investigation, with PFS being the primary outcome in two studies [18-21] and TTP [22-26], response [27], and 12-week progression-free rate [28], as the primary outcomes in one study each. There was no primary endpoint identified in the remaining one study [29]. The secondary outcome measures evaluated across these studies included OS, response, DOR, TTR, dose reductions/interruptions, safety, and withdrawals.

**Table 1:** Summary of relevant randomised controlled trials included in the review

**Interventions**	**Study**	**Study design**	N*	**Age median (range)**	**Males (%)**	**Median duration of follow-up (weeks)**	**Prior therapy for advanced disease**	**Performance status, n (%)**	**STS subtypes, (%)**
Pazopanib 800 mg per day orally	PALETTE study 2011 [18,19,30]	R, DB, PC, MC-I, Phase III	246	56.0 (20.0-83.0)	40.0%	49.5 weeks	Anthracycline: 98.8%; ifosfamide: 66.7%	PS 0: 118 (48.0); PS 1: 128 (52.0)	Leiomyosarcoma: 44.3%; Synovial sarcoma: 10.2%; Others: 45.5%
Placebo	PALETTE study 2011 [18,19,30]		123	51.0 (18.0-78.0)	44.0%	45.3 weeks	Anthracycline: 98.4%; ifosfamide: 75.6%	PS 0: 60 (48.8); PS 1: 67 (51.2)	Leiomyosarcoma: 39.8%; Synovial sarcoma: 10.6%; Others: 49.6%
Trabectedin 1.5 mg/m2 24-hour infusion q3w	Demetri 2009 [22-26]~	R, OL, DR, MC-I, Phase II	136	53 (20–80)	32.4%	177.67 weeks	Anthracycline: 100%; anthracycline and ifosfamide: 99.3%	PS 0: 70 (51.5); PS 1: 66 (48.5)	Leiomyosarcoma: 61.5%; Liposarcoma: 25.6%; Others: 12.8%
Trabectedin 0.58 mg/m2 3-hour infusion qw	Demetri 2009 [22-26]~		134	54 (23–77)	41.8%			PS 0: 67 (50.0); PS 1: 67 (50.0)	Leiomyosarcoma: 55.5%; Liposarcoma: 37.8%; Others: 6.7%
Gemcitabine 1800 mg/m2 as a fixed dose infusion rate (10 mg/m2/minutes) + dacarbazine 500 mg/m2 q2w	GEIS study [20,21]~	R, BU, AC, MC, Phase II	59	49 (18–78)	53.0%	62.83 weeks	Out of total eligible population of 109 patients 107 patients had received anthracycline and two patients had received ifosfamide	PS 0:22 (38.6); PS 1:30 (52.6); PS 2:5 (8.8)	Leiomyosarcoma: 28.1%; Liposarcoma/adipocytic sarcoma: 17.5%; Undifferentiated pleomorphic: 19.3%; Miscellaneous sarcoma: 24.6%; Synovial sarcoma: 10.5%
Dacarbazine 1200 mg/m2 q3w	GEIS study [20,21]~		54	51 (25–73)	54.0%	60.67 weeks		PS 0: 17 (32.7); PS 1: 31 (51.6); PS 2: 4 (7.7)	Leiomyosarcoma: 30.8%; Liposarcoma/adipocytic sarcoma: 17.3%; Undifferentiated pleomorphic: 15.4%; Miscellaneous sarcoma: 26.9%; Synovial sarcoma: 9.6%
Gemcitabine 900 mg/m2 over 90 minutes, D1+D8 + docetaxel 100 mg/m2 over 60 min, D8 q21 days	Pautier 2009 [27,31]~	R, BU, AC, MC, Phase II	84**	-	-	Unclear	Anthracycline: 100%	-	Leiomyosarcoma: 100%
Gemcitabine 1000 mg/m2 over 100 minutes, d1+d8+d15 q28 days	Pautier 2009 [27,31]~			-	-		Anthracycline: 100%	-	Leiomyosarcoma: 100%
Sorafenib 400 mg twice daily orally	Pacey 2011 [28]	R, DB, PC, MC-I, Phase II	2	-	-	Unclear	Anthracycline and/or ifosfamide: 100%	-	Fibrosarcoma: 0.0%
Placebo	Pacey 2011 [28]		2	67 (62–72)	0			PS 0: 2 (100)	Leiomyosarcoma: 50.0%; Fibrosarcoma: 50.0%
								PS 1: 0 (0.0)	
Ifosfamide 5 g/m2/1 day given as 24-hour infusion; all cycles were repeated q3w	van Oosterom 2002 [29]	R, BU, DR, MC-I, Phase II	27	-	-	Unclear	Anthracycline: 100%	-	-
Ifosfamide 3 g/m2/day given over 4 hour on 3 consecutive days; all cycles were repeated q3w	van Oosterom 2002 [29]		31	-	-		Anthracycline: 100%	-	-

AC: Active-controlled; BU: Blinding Unclear; DB: Double-blind; DR: Dose Ranging; ECOG: Eastern Cooperative Oncology Group; q3w: Every Three Weeks; q2w: Every Two weeks; qw: Every week; MFH: Malignant Fibrous Histiocytoma; min: Minutes; MC: Multicentre; MC-I: Multicentre International; OL: Open Label; PS: Performance Status; STS: Soft Tissue Sarcoma; *N represents number of patients randomised except for Pacey 2011 study and van Oosterom 2002 where N represents the patient population of interest with respect to prior treatment for advanced disease; **Represents total number of patients randomised in the study (number of patients randomised to each arm not reported); -Represents data not reported; ¶Represents data for the complete study population; ~Represents secondary reference.

The number of patients randomised across all RCTs was greater than 50, except for the study by Pacey and colleagues that randomised five patients [28]. Of the five patients randomised in this study, one patient was chemotherapy-naive and hence did not meet the inclusion criteria of the review. In addition, this small-sized study was not a true RCT [28]. All the patients initially received sorafenib in a 12-week open-label run-in period following which patients with ≥25% tumour shrinkage continued sorafenib, those with ≥25% tumour growth discontinued, and the remaining patients were randomised to treatment with sorafenib (2 patients) or placebo (2 patients) [28]. In terms of the patient population recruited across these studies, leiomyoscaroma was the most commonly enrolled subtype of STS followed by liposarcoma and undifferentiated pleomorphic sarcoma. Across all the included RCTs, at least 90% of patients received prior treatment in an advanced setting, except for the study by van Oosterom and colleagues [29]. This study recruited a mixed population of patients previously treated in an adjuvant or advanced setting, with limited subgroup data for the patients previously treated in the advanced setting [29].

The quality assessment of the included RCTs using the comprehensive critical appraisal tool based on the NICE and Cochrane’s critical appraisal tool is detailed in an additional file (see Additional file 3). None of the RCTs included in the review were identified as being at a high risk of bias.

#### Efficacy/activity results

Table 2 summarises the various efficacy/activity results observed across the included RCTs. The RCTs included in the review have been examined separately according to the phase of the trial.

**Table 2:** Summary of various efficacy/activity outcomes observed across randomised controlled trials

**Intervention**	**Study**	**N**	**Progression free survival**			**Overall survival**		**Response rate**				**Progressive disease**
			**PFS rate, n (%)**		**PFS in months**	**1 year OS**	**OS in months**	**ORR**	**CR**	**PR**	**SD**	**PD**
			**3-month**	**6-month**	**median (95% CI)**	**n (%)**	**median (95% CI)**	**n (%)**	**n (%)**	**n (%)**	**n (%)**	**n (%)**
Pazopanib	PALETTE study 2011/2	246	-	-	4.6†	-	12.6	11 (4.5) †	0 (0.0) †	11 (4.5) †	134 (54.5)†	66 (26.8) †
Placebo	PALETTE study 2011	123	-	-	1.6†	-	10.7	0 (0.0) †	0 (0.0) †	0 (0.0) †	33 (26.8) †	76 (61.8) †
Trabectedin 1.5 mg/m2 q3w	Demetri 2009	136	70 (51.5)	48 (35.5)	3.3† (2.1 - 4.6)	82 (60.0)	13.9 (12.5 - 18.6)	8 (5.6)†	-	-	-	-
Trabectedin 0.58 mg/m2 qw	Demetri 2009	134	60 (44.7)	37 (27.5)	2.3† (2.0 - 3.4)	67 (50.0)	11.8 (9.9 - 14.9)	2 (1.6)†	-	-	-	-
Dacarbazine	GEIS study	54	19 (35.2); p=0.001	-	2.0#	-	8.2§	2 (3.7)‡, p=0.16	-	2 (4.0)*#	10 (19.0)*#	-
Gemcitabine + Dacarbazine	GEIS study	59	32 (54.2); p=0.001	-	4.2#	-	16.8§	7 (11.9)‡, p=0.16	-	5 (9.0)* #	22 (38.0)*#	-
Sorafenib	Pacey 2011	2	-	-	-	-	-	-	-	-	-	0 (0.0) #
Placebo	Pacey 2011	2	-	-	-	-	-	-	-	0 (0.0) #	2 (100) #	0 (0.0) #
Gemcitabine	Pautier 2009	-	-	-	-	-	-	-	-	-	-	-
Gemcitabine + Docetaxel	Pautier 2009	-	-	-	-	-	-	-	-	-	-	-
Ifosfamide 5 g/m^2^/day	van Oosterom 2002	27	-	-	-	-	-	-	-	-	-	-
Ifosfamide 3 g/m^2^/day	van Oosterom 2002	31	-	-	-	-	-	-	-	-	-	-

CI: Confidence Interval; CR: Complete Response; INV: Investigator; IRC: Independent Review Committee; N: Number of evaluable Patients; n: Number with Outcome; ORR: Overall Response Rate; OS: Overall Survival; PFS: Progression-free Survival; PR: Partial Response; q3w: Every Three Weeks; qw: Every Week; SD: Stable Disease; *p=0.01; †Assessments were made by the independent review committee; ‡Assessments were made by the investigator; #Unclear if assessed by investigator or the Independent Review Committee; §Kaplan-Meier estimates reported; -Represents data not reported.

#### Phase III trials

The only Phase III trial included in the review was the PALETTE trial evaluating pazopanib (N=246) versus placebo (N=123) in advanced STS patients (excluding GIST, liposarcoma and other subtypes). The data presented here are from an analysis conducted by the manufacturer for regulatory purposes [18,19] and differ slightly from an analysis conducted by the study’s collaborative partner [30,32] as a consequence of small differences in censoring rules and data handling. A summary of these minor differences in results between analyses can be found in Additional file 4. This trial demonstrated a significantly prolonged primary endpoint of PFS (per independent review) for pazopanib compared with placebo (Hazard Ratio (HR): 0.35 [95% CI: 0.26 - 0.48]; p<0.001) [18,19]. The benefit in PFS was consistently observed across all three histological sub-types included in the study (leiomyosarcoma [p<0.001], synovial sarcoma [p=0.005], and other STS sub-types [p<0.001]). The best overall response based on the independent radiology review also favoured pazopanib. However, there was no statistically significant difference between pazopanib and placebo for median OS (HR: 0.87 [95% CI: 0.67 - 1.12]; p=0.256) [18,19]. These results should be interpreted in view of the fact that patients treated with pazopanib and placebo received post-study therapy including trabectedin (25% vs. 32%), gemcitabine (17% vs. 23%), a taxane (10% vs. 18%) and ifosfamide (10% vs. 17%) that might have potentially confounded the OS results [19]. This was the only study to report quality of life data. Based on the EORTC QLQ-C30 questionnaire, no clinically meaningful or statistically significant differences in global health status were observed between pazopanib and placebo patients remaining on treatment at the assessment time points [18,19].

#### Phase II trials

PFS rate at 3 months was the primary activity measure in the GEIS study comparing the combination of gemcitabine and dacarbazine (N=59) against dacarbazine monotherapy (N=54) [20,21]. The PFS rate at 3 months was significantly better for gemcitabine plus dacarbazine than dacarbazine monotherapy (p=0.001). Similar results, favouring the combination, were observed in terms of the secondary efficacy endpoints evaluated including median PFS (HR: 0.58 [95% CI: 0.39 - 0.86]; p=0.005), median OS (HR: 0.56 [95% CI: 0.36 - 0.90]; p=0.014), and response rate [20,21]. Fifty-three percent of patients initially treated with dacarbazine monotherapy and 51% of patients treated with the combination of gemcitabine and dacarbazine received post-study therapy comprising mainly gemcitabine-based regimens, trabectedin, and taxanes [20,21].

TTP was the primary activity endpoint in the study by Demetri and colleagues evaluating the two dosing schedules of trabectedin [22-26]. Median TTP favoured the trabectedin q3w 24-hour dosing schedule (N=136) over the qw 3-hour dosing schedule (N=134) when assessment was made by investigator (4.2 months vs. 2.5 months; HR: 0.668 [95% CI: 0.506 – 0.883]; p=0.0042) and IRC (3.7 months vs. 2.3 months; HR: 0.734 [95% CI: 0.554 - 0.974]; p=0.03) [22-26]. In terms of the secondary activity measures, median PFS was significantly longer with the q3w 24-hour schedule than the qw 3-hour schedule (p=0.0418), while no significant differences between the two dosing schedules were observed in median OS (HR: 0.843 [95% CI: 0.653 - 1.090]; p=0.1920) [22-26]. The PFS rate, 1-year OS rate, and ORR also favoured the q3w 24-hour dosing schedule over the qw 3-hour dosing schedule. Forty-nine patients in this study received post-study therapy by crossing-over to the other schedule (35 patients crossed-over after progression as allowed by the protocol [29 patients from qw 3-hour arm to q3w 24-hour arm, and 6 patients from q3w 24-hour arm to qw 3-hour arm] and 14 patients before progression [following independent data monitoring committee recommendation, all from qw 3-hour to q3w 24-hour arm]) [22-26].

Limited activity data were obtained from the remaining three RCTs included in the review [27-29]. The study by van Oosterom and colleagues evaluating two different ifosfamide regimens provided no subgroup efficacy data including OS, TTP, PFS, and response duration specifically for patients previously treated in an advanced setting [29]. Similarly, in the study by Pacey and colleagues, although the PFS rate at 12 weeks was the primary activity endpoint, the study did not report data for patients randomised to sorafenib or placebo. The only activity data reported in this study was SD in all four patients receiving sorafenib or placebo at 12 weeks [28]. Activity data were also not reported in the conference abstract for the TAXOGEM study by Pautier and colleagues [27].

#### Safety results

Overall, AEs were not consistently reported across the RCTs included in the review. The most commonly reported grade 3/4 AEs (≥5%) in association with pazopanib in the Phase III PALETTE trial were fatigue (14%), lymphopenia (10%) tumour pain (8%), increased alanine transaminase (ALT) (10%), increased aspartate aminotransferase (AST) (8%), hypertension (7%), dyspnoea (6%), anaemia (6%), decreased appetite (6%), and diarrhoea (5%) [18,19]. Across the Phase II trials, haematological AEs were commonly experienced with treatments including dacarbazine, gemcitabine plus dacarbazine, and trabectedin. In addition to haematological AEs, ≥5% of patients experienced grade 3/4 ALT increase, creatinine phosphokinase increase, and fatigue with the two dosing schedules of trabectedin [22-26] and grade 3/4 asthenia with gemcitabine plus dacarbazine and dacarbazine monotherapy [20,21]. Grade 3/4 nausea, vomiting, and AST increase were also experienced by ≥5% of patients treated with trabectedin 24-hour schedule [22-26]. Summaries for grade 3 and/or 4 AEs reported in >1% patients across the included studies are shown in Table 3.

**Table 3:** Summary of grade 3 and/or 4 specific adverse events reported in >1% patients across randomised controlled trials

**AEs by class**	**PALETTE study 2011**^**#**^		**Demetri 2009**		**GEIS study 2011**		**Pautier 2009**		Pacey 2011*		**van Oosterom 2002**	
	**Pazopanib, n (%)**	**Placebo, n (%)**	**Trabectedin 1.5 mg/m2 q3w, n (%)**	**Trabectedin 0.58 mg/m2 qw, n (%)**	**Dacarbazine, n (%)**	**Gemcitabine + Dacarbazine, n (%)**	**Gemcitabine, n (%)**	**Gemcitabine + Docetaxel, n (%)**	**Placebo, n (%)**	**Sorafenib, n (%)**	**Ifosfamide 5 g/m2/day, n (%)**	**Ifosfamide 3 g/m2/day, n (%)**
**Evaluable N**	**240**	**123**	**130**	**130**	**52**	**57**	**-**	**-**	**2**	**2**	**27**	**31**
**GI disorders**												
Abdominal pain	0 (0.0)	0 (0.0)	6 (4.6)	6 (4.6)	-	-	-	-	-	-	-	-
Constipation	1 (0.4)	3 (2.4)	0 (0.0)	2 (1.5)	-	-	-	-	-	-	-	-
Diarrhoea	11 (4.6)	1 (0.8)	1 (0.8)	0 (0.0)	0 (0.0)	0 (0.0)	-	-	-	-	-	-
GI pain	6 (2.5)	5 (4.1)	-	-	-	-	-	-	-	-	-	-
Mucositis/stomatitis	1 (0.4)	0 (0.0)	-	-	0 (0.0)	1 (1.8)	-	-	-	-	-	-
Nausea	8 (3.3)	2 (1.6)	7 (5.4)	3 (2.3)	1 (1.9)~	0 (0.0)~	-	-	-	-	-	-
Nausea/vomiting	-	-	-	-	-	-	-	-	-	-	0 (0.0)$	-
Vomiting	8 (3.3)	1 (0.8)	7 (5.4)	2 (1.5)	0 (0.0)~	1 (1.8)~	-	-	-	-	-	-
Small intestinal obstruction	3 (1.3)	0 (0.0)	-	-	-	-	-	-	-	-	-	-
**General disorders**												
Asthenia	0 (0.0)	0 (0.0)	-	-	5 (9.6)~	4 (7.0)~	-	-	-	-		-
Back pain	-	-	4 (3.1)	4 (3.1)	-	-	-	-	-	-	-	-
Fatigue	33 (13.7)	6 (4.9)	10 (7.7)	9 (6.9)	-	-	-	-	-	-	-	-
Peripheral oedema	5 (2.1)	2 (1.6)	-	-	-	-	-	-	-	-	-	-
Chest pain	4 (1.7)	0 (0.0)										
**Skin and subcutaneous tissues disorders**												
Skin disorder	4 (1.7)	0 (0.0)	-	-	-	-	-	-	-	-	-	-
**Investigations**												
ALT increased	23 (9.6)	4 (3.3)	62 (47.7)	12 (9.2)	-	-	-	-	0 (0.0)	0 (0.0)	-	-
Alkaline phosphatase	7 (2.9)	1 (0.8)	2 (1.5)	3 (2.3)	-	-	-	-	-	-	-	-
AST increased	19 (7.9)	2 (1.6)	41 (31.5)	4 (3.1)	-	-	-	-	0 (0.0)	0 (0.0)	-	-
Creatinine increased	1 (0.4)	0 (0.0)	3 (2.3)	1 (0.8)	-	-	-	-	-	-	-	-
Creatinine phosphokinase	-	-	7 (5.4)	12 (9.2)	-	-	-	-	-	-	-	-
Bilirubin increased	3 (1.3)	2 (1.6)	1 (0.8)	1 (0.8)	-	-	-	-	0 (0.0)	0 (0.0)	-	-
Gamma-glutamyltransferase	4 (1.7)	0 (0.0)	-	-	-	-	-	-	-	-	-	-
Weight loss	9 (3.8)	0 (0.0)	-	-	-	-	-	-	0 (0.0)	0 (0.0)	-	-
ENT examination abnormal	4 (1.7)	0 (0.0)	-	-	-	-	-	-	-	-	-	-
Hypoalbuminemia	-	-	-	-	-	-	-	-	0 (0.0)	0 (0.0)	-	-
**Hemorrhagic events**												
Hemorrhagic event (any)	5 (2.0)	2 (1.6)	-	-	-	-	-	-	0 (0.0)	0 (0.0)	-	-
**Metabolism and nutrition disorders**												
Decreased appetite	14 (5.9)	0 (0.0)	1 (0.8)	0 (0.0)	-	-	-	-	-	-	-	-
Dehydration	3 (1.4)	0 (0.0)	-	-	-	-	-	-	-	-	-	-
**Musculoskeletal and connective tissue disorders**												
Arthralgia/myalgia	5 (2.1)	0 (0.0)	-	-	-	-	-	-	0 (0.0)	0 (0.0)	-	-
Musculoskeletal pain	5 (2.1)	2 (1.6)	-	-	-	-	-	-	-	-	-	-
**Respiratory, thoracic, and medistinal disorders**												
Dyspnoea	15 (6.3)	7 (5.7)	5 (3.8)	8 (6.2)	-	-	-	-	-	-	-	-
Pleural effusion	5 (2.1)	0 (0.0)	-	-	-	-	-	-	-	-	-	-
**Blood and lymphatic system disorders**												
Anaemia	15 (6.3)	2 (1.6)	10 (7.7)	12 (9.2)	6 (11.5)	2 (3.5)	-	-	-	-	-	-
Febrile neutropenia	-	-	-	-	3 (5.8)	5 (8.8)	-	-	-	-	-	-
Leukopenia	3 (1.3)	0 (0.0)	-	-	16 (30.8)	15 (26.3)	-	-	-	-	-	-
Neutropenia	10 (4.2)	0 (0.0)	61 (46.9)	17 (13.1)	17 (32.7)	27 (47.4)	-	-	-	-	-	-
Thrombocytopenia	9 (3.8)	0 (0.0)	15 (11.5)	7 (5.4)	14 (26.9)	3 (5.3)	-	-	-	-	0 (0.0)$	-
Lymphopenia	23 (9.6)	13 (10.6)	-	-	-	-	-	-	-	-	-	-
**Cardiac disease**												
Hypertension	16 (6.7)	0 (0.0)	-	-	-	-	-	-	-	-	-	-
Myocardial/LVEF Dysfunction	4 (1.7)	0 (0.0)	-	-	-	-	-	-	-	-	-	-
**Other disorders**												
Tumour pain	20 (8.3)	9 (7.3)										
Non-haemotological	-	-	-	-	-	-	-	2¶	-	-	-	-

The data for the PALETTE study is likely to be more comprehensive than for the other studies since extracted from the CSR versus published reports for the other studies; AE: Adverse Events; ALT: Alanine Transaminase; AST: Aspartate Aminotransferase; GI: Gastrointestinal; N: Number of Patients; n: Number with Outcome; q3w: Every Three Weeks; qw: Every Week; *Grade 3–5 AEs are reported in Pacey 2011 trial; #Data reported for haematology and liver enzyme abnormalities in PALETTE study by maximum grade shift (any increase in grade) based on clinical laboratory evaluations; all other data for PALETTE based on AE reports; -Represents data not reported; $Represents Grade 4 event; ~Represents Grade 3 event; ¶ Number of patients analysed was not reported and hence, percentage of patients could not be computed.

#### Treatment discontinuations

Overall, four of the six included RCTs reported data related to treatment discontinuations. In the Phase III PALETTE trial, pazopanib was associated with a higher proportion of patients discontinuing treatment due to AEs compared with placebo (Table 4) [19]. Across the Phase II RCTs, the proportion of patients discontinuing treatment due to AEs were comparable with the two dosing schedules of trabectedin [22-26], while in the GEIS study none of the patients treated with gemcitabine plus dacarbazine discontinued therapy due to AEs [20,21]. Summaries of the treatment discontinuations observed across the included RCTs are shown in Table 4.

**Table 4:** Results of treatment discontinuations across randomised controlled trials

**Intervention**	**Study**	**N**	**Treatment discontinuation, n (%)**							**Most common AEs leading to discontinuations**
			**All**	**Due to AE**	**Due to death**	**Due to PD**	**Due to lost to follow-up**	**Due to patient decision**	**Due to other reasons**	
Pazopanib	PALETTE study 2011/2	246	240 (97.6)	41 (16.7)	3 (1.2)	178 (72.4)	0 (0.0)	14 (5.7)	4 (1.6)¶	ALT elevation, dyspnoea, left ventricular dysfunction, fatigue, hypertension, vomiting, depressed mood, embolism, nausea, pericardial effusion, and small intestinal obstruction
Placebo	PALETTE study 2011/2	123	123 (100)	3 (2.4)	0 (0)	119 (96.7)	0 (0.0)	1 (0.8)	0 (0.0)	Dyspnoea
Trabectedin 1.5 mg/m2 q3w	Demetri 2009	136	128 (94.1)	12 (8.8)	2 (1.5)	89 (65.4)	2 (1.5)	17 (12.5)	6 (4.4)†	AEs leading to disconsolation were not reported
Trabectedin 0.58 mg/m2 qw	Demetri 2009	134	134 (100)	10 (7.5)	3 (2.2)	94 (70.1)	0 (0.0)	6 (4.5)	21 (15.7)‡	AEs leading to disconsolation were not reported
Dacarbazine	GEIS study 2011	54	50 (92.6)	2 (3.7)	-	41 (75.9)	0 (0.0)	-	7 (13.0)	-
Gemcitabine + Dacarbazine	GEIS study 2011	59	47 (79.7)	0 (0.0)	-	36 (61.0)	0 (0.0)	1 (1.7)	10 (16.9)	-
Gemcitabine	Pautier 2009	84*	-	3	-	-	-	-	-	AEs leading to disconsolation were not reported
Gemcitabine + Docetaxel	Pautier 2009		-	9	-	-	-	-	-	Hypersensitivity in one patient

AE: Adverse Events; N: Number of Patients; n: Number with Outcome; PD: Progressive Disease; q3w: Every Three Weeks; qw: Every Week; -Represents data not reported; *Represents total population randomised in the study (patients randomised in each arm not reported); ¶Includes three patients discontinuing treatment due to protocol violation; †Includes patients discontinuing due to screening failure (n=3) and ineligibility of patients (n=2); ‡Includes patients discontinuing due to screening failure (n=2), cross-over before progression (n=14), and ineligibility of patients (n=1).

### Non-randomised studies

#### Trial characteristics

A summary of the 52 prospective non-randomised studies with sample size more than 10 [33-72] is presented in Table 5. Further details regarding the study design and patient characteristics for these studies are presented in an additional file (see Additional file 5). The list of retrospective studies and studies with a sample size less than 10 is also provided as an additional file (see Additional file 6).

**Table 5:** Summary of relevant prospective non-randomised trials (with sample size more than 10) included in the review

**Intervention**	**Dose**	**Study**	**N**	**STS subtypes (%)**
Brostallicin	10 mg/m^2^	Leahy 2007 [33]	43	Multiple
Cisplatin	50 mg/m^2^	Thigpen 1986 [34]	20	Leiomyosarcoma
Cyclophosphamide	1.5 g/m^2^	Bramwell 1993 [35,36]~	18	NS
Dacarbazine	1200 mg/m^2^	Buesa 1991 [73]	47	Multiple
Docetaxel	100 mg/m^2^	Kostler 2001 [37]	25	Multiple
Docetaxel	100 mg/m^2^	Santoro 1999 [38]	37	Multiple
Docetaxel	100 mg/m^2^	van Hoesel 1994 [39,40]~	21	NS
Doxorubicin	75 mg/m^2^	Mouridsen 1987 [41]	23	NS
Etoposide	200-240 mg/m^2^/day	Crawley 1997 [42]	17	Multiple
Etoposide	130 mg/m^2^	Dombernowsky 1987 [43]	26	NS
Gefitinib	500 mg	Ray-Coquard 2008 [44]	48	Synovial sarcoma≈
Gemcitabine	1000 mg/m^2^	Ferraresi 2008 [74]	14	Multiple
Gemcitabine	1000 mg/m^2^	Hartmann 2006 [45]	15	Multiple
Gemcitabine	1000 mg/m^2^	Look 2004 [75]	35	Leiomyosarcoma
Gemcitabine	200-250 mg/m^2^	Spath-Schwalbe 2000 [46]	18	Multiple
Ifosfamide	2 g/m^2^ for 4d q3w	Antman 1985 [47]	31	Multiple
Ifosfamide	2 g/m^2^ for 4d q3w	Antman 1989 [48]	94	Multiple
Ifosfamide	1 g/m^2^ daily until Gr3 granulocytopenia q4w	Babovic 1998 [76]	21	Multiple
Ifosfamide	4 g/m^2^ for 3d q4w	Le Cesne 1995 [49,77]~	40	Multiple
Ifosfamide	4 g/m^2^ for 3d q4w	Nielsen 2000 [78]	13	NS
Ifosfamide	3.5 g/m^2^ for 4d q3w	Palumbo 1997 [79]	38	Multiple
Ifosfamide	4 g/m^2^ for 3.5d q3w	Patel 1997 [80]	12	Multiple
Ifosfamide	2g/m^2^ loading dose followed by 4g/m^2^ for 3d q3w	Patel 1997 [80]	32	Multiple
Ifosfamide	60 mg/kg for 5d q3-4w	Scheulen 1983 [81]	16	NS
Liposomal doxorubicin	55 mg/m^2^	Skubitz 2003 [50]	20	Multiple
Liposomal doxorubicin	30-50 mg/m^2^	Toma 2000 [51]	25	Multiple
Methotrexate	40 mg/m^2^	Buesa 1984 [52]	37	NS
Paclitaxel	135-175 mg/m^2^	Palumbo 1997 [53]	12	Multiple
Paclitaxel	200 mg/m^2^	Patel 1997 [54]	12	Multiple
Paclitaxel	120 mg/m^2^	Skubitz 1997 [55]	17	Multiple
Sorafenib	400 mg	Bertuzzi 2010 [56,57]~	61	Multiple
Sorafenib	400 mg	Pacey 2011 [28]	16	Multiple
Sunitinib	50 mg	Decoster 2010 [58]	24	NS
Trabectedin	1.5 mg/m^2^	Garcia-Carbonero 2004 [59,60,82]~	36	Multiple
Trabectedin	1.5 mg/m^2^	Le Cesne 2005 [83]	104	Multiple
Trabectedin	1.5 mg/m^2^	Yovine 2004 [61,84]~	27	Multiple
Biricodar + doxorubicin	B - 120 mg1.5 mg/m^2^/hr; D – 60 mg/m^2^	Bramwell 2002 [62]	18	Multiple
Cisplatin + ifosfamide	C – 100 mg/m^2^ day 2&9; I −2.5 g/m^2^ for 3d	Budd 1993 [85]	38	Multiple
Cisplatin + vinblastine	C – 50–100 mg/m^2^ day 1; V – 1.0-1.2 mg/m^2^ for 5d	Keohan 1997 [63]	18	Multiple
D + IL-2	D – 70 mg/m^2^; IL-2 - 18 MIU/m^2^	Le Cesne 1999 [64]	12	NS
Epirubicin + lonidamine	E – 120 mg/m^2^; I – 150–450 mg	Lopez 1995 [65]	25	Multiple
Etoposide + ifosfamide	E – 200 mg/m^2^ for 3d; I – 1.5 g/m^2^ for 3d	Saeter 1995 [86]	11	NS
Etoposide + ifosfamide	E – 50 mg/m^2^ for 8d; I - 1.5 g/m^2^ for 6d	Skubitz 1993 [87]	16	Multiple
Gemcitabine + dacarbazine	G – 800–2160 mg/m^2^; DTIC – 500 mg/m^2^	Buesa 2004 [66]	22	Multiple
Gemcitabine + dacarbazine	G – 1800 mg/m^2^; DTIC – 500 mg/m^2^	Losa 2007 [67]	26	Multiple
Gemcitabine + docetaxel	G – 900 mg/m^2^; D – 100 mg/m^2^	Hensley 2002 [88]	16	Leiomyosarcoma
Gemcitabine + docetaxel	G – 900 mg/m^2^; D – 100 mg/m^2^	Hensley 2008 [89]	51	Leiomyosarcoma
Gemcitabine + docetaxel	G – 900 mg/m^2^; D – 100 mg/m^2^	Montalar 2008 [68]	12	Multiple
Methotrexate + vincristine	M – 5 g/m^2^; V – 1 mg/m^2^	Vaughn 1984 [69]	14	NS
VAC + IE	V – 2 mg; A – 70 mg/m^2^; C – 600 mg/m^2^; I – 1.8 g/m^2^; E – 500 mg/m^2^	Palumbo 1998 [70]	12	NS
C + VC + D + DTIC + IL-2	IL-2 – 18 m units/d, followed by CYVADIC	Gravis 2001 [71]	1	Leiomyosarcoma
D + I + DTIC + IL-2	IL-2 – 18 m units/d, followed by D+I+DTIC	Gravis 2001 [71]	9	Multiple
D + IL-2	IL-2 – 18 m units/d, followed by D	Gravis 2001 [71]	3	Multiple
Carboplatin + etoposide	C – 300 mg/m^2^; E – 300 mg/m^2^	Holstein 1996 [72]	8	Multiple
Dacarbazine	1200 mg/m^2^	Holstein 1996 [72]	14	Multiple

C + VC + D + DTIC + IL-2: Cyclophosphamide + Vincristine + Doxorubicin + Dacarbazine + Interleukin-2; D + IL-2: Doxorubicin + Interleukin-2; D + I + DTIC + IL-2: Doxorubicin + Ifosfamide + Dacarbazine + Interleukin-2; N: Number of Included Patients; n-RCT: Non-Randomised Controlled Trials; VAC + IE: Vincristine + Adriamycin + Cyclophosphamide + Ifosfamide + Etoposide; ~Represents secondary reference; NS: Not Specified; ≈Comprise 4.2% other soft tissue sarcoma.

The majority of the included prospective studies were Phase II trials with a variety of chemotherapeutic regimens evaluated across these studies. Ifosfamide was the most commonly evaluated monotherapy (nine studies) followed by gemcitabine (four studies), docetaxe l (three studies), paclitaxel (three studies), and trabectedin (three studies), while gemcitabine-based regimens were the most frequently evaluated combination therapy (five studies). Response, PFS, DOR, TTP, OS, and safety were the most commonly assessed outcomes in the included studies.

The quality assessment of the included non-randomised studies based on the Downs and Black checklist demonstrated that studies were reported reasonably well in terms of study question, methods, patient population, outcomes measures, and results [16].

#### Efficacy results

Across the non-randomised evidence, there was heterogeneity in the efficacy results. For example, in nine studies assessing ifosfamide monotherapy, variable activity was observed in terms of response rate (4.8% [76] to 62.5% [81]) (Table 6). Although a wide variety of doses and schedules was used in these trials, with cumulative dose per cycle ranging from 8 to 14 mg/m^2^, this did not have a clear impact on efficacy. Gemcitabine as monotherapy (four studies) and in combination therapy demonstrated a variable efficacy in terms of median OS (monotherapy: 6.0 months to 11.8 months; combination therapy: 14.7 months) and ORR (monotherapy: 6.7% to 21.2%; combination therapy: 3.8% to 50.0%). However, a superior response rate (>20%) was observed with gemcitabine monotherapy (21.2% vs. 6.7% to 11.1%) and gemcitabine plus docetaxel (25.5% to 50.0% vs. 8.3%) among patients with uterine leiomyosarcoma compared with patients with mixed STS subtypes (Table 6). Once more, there were no clear trends relating to dose of gemcitabine.

**Table 6:** Efficacy/Activity outcomes across prospective non-randomised trials with sample size more than 10

**Intervention**	**Study**	**N**	**Response rate n (%), IRC or unclear**					**PFS**	**TTP in months**	**OS**	**DOR in months**	**Progressive disease**
			**ORR**	**CR**	**PR**	**MR**	**SD**	**n (%) or median (95% CI)**	**Median (range)**	**n (%) or median (range)**	**Median (range)**	**n (%), IRC or unclear**
Brostallicin	Leahy 2007	40	2 (5.0%)~	0 (0.0 %)~	2 (5.0%) ~	-	20 (50.0 %)~	3 mo: 18 (45.0%)~; 6 mo: 9 (22.5%)^~^	2.9 months~ (1.4 - 3.7)	7.6 months (5.2 - 13.8)	-	17 (42.5%)~
Cisplatin	Thigpen 1986	19	1 (5.3%)	1 (5.3%)	0 (0.0%)	-	7 (36.8%)	-	-	-	9 months (N = 1)	11 (57.9%)
Cyclophosphamide	Bramwell 1993	18	0 (0.0%)	0 (0.0%)	0 (0.0%)	-	-	-	-	-	-	-
Dacarbazine	Buesa 1991	44	8 (18.2%)	1 (2.3%)	7 (15.9%)	-	8 (18.2%)	-	-	-	-	28 (63.6%)
Docetaxel	Kostler 2001	25	4 (16.0%)	0 (0.0%)	4 (16.0%)	-	-	-	-	-	-	-
Docetaxel	Santoro 1999	36	1 (2.8%)#	0 (0.0%)	1 (2.8%)	-	10 (27.8%)	1.4 months (N = 37)	-	11.5 months (N = 37)	-	25 (69.4%)
Docetaxel	Van Hoesel 1994	21	-	0 (0.0%)~	-	-	-	-	-	-	-	-
Doxorubicin	Mouridsen 1987	23	2 (8.7%)~	-	-	-	-	-	-	-	-	-
Etoposide	Crawley 1997	16	0 (0.0%)	0 (0.0%)	0 (0.0%)	-	8 (50.0%)	-	-	3.7 months (2.1 - 5.2)* (N = 17)	-	8 (50.0%)
Etoposide	Dombernowsky 1987	26	-	-	1 (3.8%)	-	-	-	-	-	19 months (N = 1)	-
Gefitinib	Ray-Coquard 2008	46	0 (0.0%)~	0 (0.0%)~	0 (0.0%)~	0 (0.0%)~	10 (21.7%)~	4 mo: 10 (21.7%)^~^; 6 mo: 3 (6.5%)^~^	1.4 months~	-	-	32 (69.6%)~
Gemcitabine	Ferraresi 2008	14	1 (7.1%)	0 (0.0%)	1 (7.1%)	-	3 (21.4%)	-	3.1 months (1.0 - 9.5)	11.8 months (1.0 - 54.5)	6.5 months (N = 1)	10 (71.4%)
Gemcitabine	Hartmann 2006	15	1 (6.7%)	0 (0.0%)	1 (6.7%)	-	7 (46.7%)	3 mo: 7 (46.7%); 6 mo: 2 (13.3%); 3.0 months (1.0 – 33.0)	3 months	6.0 months (3.0 - 33.0)	-	7 (46.7%)
Gemcitabine	Look 2004	33	7 (21.2%)	1 (3.0%)	6 (18.2%)	-	-	-	-	-	-	-
Gemcitabine	Spath-Schwalbe 2000	18	2 (11.1%)#	0 (0.0%)	2 (11.1%)	-	6 (33.3%)	-	-	12 mo: 5 (27.8%); 8 months	5.5 months (5.0 - 6.0) (N = 2)	9 (50.0%)
Ifosfamide	Antman 1985	26	8 (30.8%)#	0 (0.0%)	8 (30.8%)	3 (11.5%)	11 (42.3%)	-	-	-	(2.0 – 10.0+) (N = 11)	4 (15.4%)
Ifosfamide	Antman 1989	94	17 (18.1%)	2 (2.1%)	-	-	-	-	-	-	-	-
Ifosfamide	Babovic 1998	21	1 (4.8%)	1 (4.8%)	0 (0.0%)	-	4 (19.0%)	-	-	-	-	16 (76.2%)
Ifosfamide	Le Cesne 1995	36	12 (33.3%)	0 (0.0%)	12 (33.3%)	-	8 (22.2%)	-	-	20.0 months (6.0 – 91.0+) (N = 40)	8.0 months (6.0 – 13.0+) (N = 12)	16 (44.4%)
Ifosfamide	Nielsen 2000	Unclear	1 (7.7%)~	0 (0.0%) ~	1 (7.7%) ~	-	-	-	-	-	-	-
Ifosfamide	Palumbo 1997a	38	15 (39.5%)	1 (2.6%)	14 (36.8%)	-	17 (44.7%)	-	-	13.0 months (6.0 – 30.0+)	9.0 months (5.0 – 21.0+) (N = 15)	6 (15.8)
Ifosfamide	Patel 1997b	11	5 (45.5%)	0 (0.0%)	5 (45.5%)	-	2 (18.2%)	-	-	-	-	4 (36.4%)
Ifosfamide	Patel 1997b	32	6 (18.8%)	2 (6.3%)	4 (12.5%)	-	-	-	-	-	-	-
Ifosfamide	Scheulen 1983	16	10 (62.5%)	2 (12.5%)	3 (18.8%)	5 (31.3%)	1 (6.3%)	-	-	-	-	5 (31.3%)
Liposomal doxorubicin	Skubitz 2003	20	1 (5.0%)#	1 (5.0%)	0 (0.0%)	3 (15.0%)	3 (15.0%)	-	-	-	-	12 (60.0%)
Liposomal doxorubicin	Toma 2000	25	3 (12.0%)	0 (0.0%)	3 (12.0%)	2 (8.0%)	17 (68.0%)	-	-	12.0 months (6.0 – 16.0+)	(3.0 – 9.0+) (N = 3)	3 (12.0%)
Methotrexate	Buesa 1984	21	0 (0.0%)	0 (0.0%)	0 (0.0%)	-	-	-	-	-	-	-
Paclitaxel	Palumbo 1997b	12	1 (8.3%)#	0 (0.0%)	1 (8.3%)	1 (8.3%)	6 (50.0%)	-	3 months	6 months	4 months (N = 1)	4 (33.3%)
Paclitaxel	Patel 1997a	12	0 (0.0%)	0 (0.0%)	0 (0.0%)	-	-	-	-	-		-
Paclitaxel	Skubitz 1997	15	1 (6.7%)#	0	1 (6.7%)	-	-	-	-	-	12 months (N = 1)	-
Sorafenib	Bertuzzi 2010	61	-	-	9 (14.8%)	-	-	6 mo: 20 (32.7%)	-	6 mo: 41 (67.2%) (N = 61)	-	-
Sorafenib	Bertuzzi 2010	30$	2 (6.7%)#	1 (3.3%)	1 (3.3%)	-	18 (60.0%)	-	-	-	-	10 (33.3%)
Sorafenib	Pacey 2011	16	-	-	-	-	0 (0.0%)	-	-	-	-	-
Sunitinib	Decoster 2010	20	1 (5.0%)#	0 (0.0%)	1 (5.0%)	-	4 (20.0%)	-	-	-	8.3 months (N =1)	15 (75.0%)
Trabectedin	Garcia-Carbonero 2004	36	3 (8.3%)	1 (2.8%)	2 (5.6%)	2 (5.6%)	-	12 mo: 4 (11.1%)	1.7 months (1.3 - 4.4)*	6 mo: 27 (75.0%); 12 mo: 19 (52.7%); 12.1 months (8.1 - 26.5)*	9.0 months (4.0 – 20.0) (N = 3)	-
Trabectedin	Le Cesne 2005	104	8 (8.7%)~ (N = 92)	0 (0.0%) ~ (N = 92)	8 (8.7%) ~(N = 92)	-	44 (47.8%) ~† (N=92)	3 mo: 54 (52%)~; 6 mo: 30 (29%)~; 9 mo: 21 (20%)~; 12 mo: 18 (17%)~	3.4 months (2.5 - 4.1)* ~ (N = 99)	12 mo: 44 (42%); 9.1 months (7.8 - 12.1)*	11.6 months~ (N = 8)	35 (38.0%)~ (N = 92)
Trabectedin	Yovine 2004	27	2 (7.4%) ~	0 (0.0%) ~	2 (7.4%) ~	2 (7.4%) ~	4 (14.8%) ~	-	-	-	12.2 months~ (N = 2)	-
Biricodar + doxorubicin	Bramwell 2002	15	2 (13.3%) ~#	0 (0.0%) ~	2 (13.3%) ~	-	7 (46.7%) ~	3.1 months~ (N = 15)	-	-	-	6 (40.0%)~
Cisplatin + ifosfamide	Budd 1993	38	8 (21.0%)	3 (7.9%)	5 (13.2%)	-	-	-	-	11 months	-	-
Cisplatin + vinblastine	Keohan 1997	15	0 (0.0%)	0 (0.0%)	0 (0.0%)	-	7 (46.7%)	-	2.0 months (0.7 - 12.0) (N =18)	-	-	8 (53.3%)
D + IL-2	Le Cesne 1999	12	2 (16.7%)	0 (0.0%)	2 (16.7%)	-	-	-	-	-	-	-
Epirubicin + lonidamine	Lopez 1995	24	2 (8.3%)	0 (0.0%)	2 (8.3%)	-	1 (4.2%)	-	-	14 months (N =24)	6.5 months (N =2)	-
Etoposide + ifosfamide	Saeter 1995	10	6 (60.0%)	0 (0.0%)	6 (60.0%)	-	-	-	-	-	-	-
Etoposide + ifosfamide	Skubitz 1993	15	6 (40.0%)	0 (0.0%)	6 (40.0%)	-	5 (33.3%)	-	-	-	(3.0 – 31.0+)~ (N = 6)	4 (26.7%)
Gemcitabine + dacarbazine	Buesa 2004	22	5 (26.3%) (N = 19)	0 (0.0%) (N = 19)	5 (26.3%) (N = 19)	-	6 (31.6%) (N=19)	3 mo: 9 (40.9%) (N = 22); 6 mo: 6 (27.3%) (N = 22)	-	-	6.5 months (2.5 – 36.0) (N =5)	8 (42.1%) (N = 19)
Gemcitabine + dacarbazine	Losa 2007	23	1 (4.3%)	1 (4.3%)	0 (0.0%)	-	11 (47.8%)	3 mo: 12 (46.2%) (N = 26); 6 mo: 7 (26.9%) (N = 26)	3.6 months (N = 23)	8.5 months (N = 23)	-	11 (47.8%)
Gemcitabine + docetaxel	Hensley 2002	16	8 (50.0%) ~	1 (6.3%) ~	7 (43.8%) ~	-	-	-	-	-	-	-
Gemcitabine + docetaxel	Hensley 2008	48	13 (27.1%)	3 (6.3%)	10 (20.8%)	-	24 (50.0%)	3 mo: 35 (72.9%) (N = 48); 6 mo: 25 (52.1%) (N = 48); 6.7 months (0.7 – 27.0+) (N = 48)	-	14.7 months (0.8 - 50.9+) (N = 48)	9.0 months (3.9 - 24.5) (N = 13)	8 (16.7%)
Gemcitabine + docetaxel	Montalar 2008	12	1 (8.3%)#	0 (0.0%)	1 (8.3%)	-	4 (33.3%)	-	-	-	-	7 (58.3%)
Methotrexate + vincristine	Vaughn 1984	14	2 (14.3%)	0 (0.0%)	2 (14.3%)	-	3 (21.4%)	-	-	-	-	-
VAC + IE	Palumbo 1998	12	3 (25.0%)	0 (0.0%)	3 (25.0%)	-	4 (33.3%)	-	-	-	-	3 (25.0%)
C + VC + D + DTIC + IL-2	Gravis 2001	1	0 (0.0%)	0 (0.0%)	0 (0.0%)	-	-	-	-	-	-	1 (100%)
D + I + DTIC + IL-2	Gravis 2001	9	0 (0.0%)	0 (0.0%)	0 (0.0%)	-	1 (11.1%)	-	-	-	-	8 (88.9%)
D + IL-2	Gravis 2001	3	1 (33.3%)	0 (0.0%)	1 (33.3%)	-	-	-	-	-	2.0 months (N = 1)	2 (66.7%)
Carboplatin + etoposide	Holstein 1996	8	0 (0.0%)#	0 (0.0%)	0 (0.0%)	-	2 (25.0%)†	-	-	12.0 months (4.0 – 25.0)	-	6 (75.0%)
Dacarbazine	Holstein 1996	14	0 (0.0%)#	0 (0.0%)	0 (0.0%)	-	2 (14.3%) †	-	-	5.0 months (1.0 – 11.0)	-	12 (85.7%)

CI: Confidence Interval; CR: Complete Response; C + VC + D + DTIC + IL-2: Cyclophosphamide + Vincristine + Doxorubicin + Dacarbazine + Interleukin-2; DOR: Duration of Response; D + IL-2: Doxorubicin + Interleukin-2; D + I + DTIC + IL-2: Doxorubicin + Ifosfamide + Dacarbazine + Interleukin-2; INV: Investigator; IRC: Independent Review Committee; mo: Months; MR: Minimal Response; mo: Months; N: Number of evaluable patients; n: Number with Outcome; ORR: Overall Response Rate; OS: Overall Survival; PFS: Progression-free Survival; PR: Partial Response; SD: Stable Disease; TTP: Time to progression; TTR: Time to Response; VAC + IE: Vincristine + Adriamycin + Cyclophosphamide + Ifosfamide + Etoposide; -Represents data not reported *95% Confidence Interval; ~Represents data assesssed by IRC; for other data it was unclear if assessed by investigator or the Independent Review Committee; #ORR calculated as CR + PR; †Represents no change; $Represents the subgroup of patients with both RECIST and CHOI evaluations for response.

The only treatment that demonstrated a similar anti-tumour activity across different trials was trabectedin (three studies). Trabectedin was associated with a response rate ranging from 7.4% [84] to 8.5% [83] and median OS varying between 9.1 months [77] and 12.1 months [82]. Limited anti-tumour activity (ORR ≤5%) in pre-treated patients with STS was demonstrated by treatments including brostallicin, cisplatin, cyclophosphamide, dacarbazine, gefitinib, methotrexate, and sunitinib [90]. Summaries of the efficacy results for interventions evaluated across non-randomised studies are presented in Table 6.

#### Safety results

Across the non-randomised studies identified in the review, haematological AEs were the most commonly reported AEs, particularly with therapies including docetaxel, gemcitabine, docetaxel plus gemcitabine, etoposide, carboplatin plus etoposide, cisplatin plus ifosfamide, and trabectedin. However, AEs were not reported in a sufficiently consistent manner for a meaningful comparison across studies. The details of the grade 3 and/or 4 AEs observed across these studies are provided in an additional file (see Additional file 7).

Data related to treatment discontinuations due to AEs were reported in only three of the included studies. Thus, it was difficult to draw any conclusions regarding the comparative tolerability of the evaluated interventions [74,82,83].

## Discussion

The objective of this systematic review was to comprehensively review available evidence on the efficacy and safety of treatments used for advanced STS following prior therapy with anthracycline- and/or ifosfamide for advanced disease. Due to the paucity of RCT evidence in this anthracyline pre-treated setting, this review included RCTs and non-randomised studies (prospective and retrospective) to allow for a detailed description of the evidence supporting these interventions being used for the management of patients with pre-treated advanced STS.

The available RCT evidence (from six studies) suggests that pazopanib, trabectedin, and the combination of gemcitabine and dacarbazine are effective treatments for pre-treated patients with advanced STS. These agents were also among those identified as potentially active second-line treatments in a recent analysis of Phase II studies by Penel and colleagues [91]. Pazopanib has demonstrated a significant advantage over placebo with an increase of 3 months in median PFS [18,19]. Treatment with q3w 24-hour dosing schedule of trabectedin was associated with significantly greater median PFS and TTP compared with the qw 3-hour schedule [22-26], and the combination of gemcitabine and dacarbazine was more effective than dacarbazine monotherapy in terms of 3-month PFS rate, median PFS, and median OS [20,21]. These findings should be interpreted in view of the fact that the evidence comes from Phase II studies except for the pazopanib PALETTE study. The primary aim of Phase II trials is to evaluate if the intervention under investigation demonstrates clinical activity and is well tolerated, and hence, they do not provide a definitive answer regarding the clinical benefit of the intervention in question. Further, post-study therapy was documented in three of the six included RCTs and this may have potentially confounded the OS results [18-20,22-26].

In view of limited RCT evidence, data from non-randomised studies was evaluated. The 52 prospective non-randomised studies included in the review suggested anti-tumour activity (3-month PFS rate ≥39%, and/or 6-month PFS rate ≥14%, [90], and/or ORR ≥10%) of several therapies including: single-agent ifosfamide [77-81] and dacarbazine [73], and that of the combinations, etoposide plus ifosfamide [86,87] and cisplatin plus ifosfamide [85]. Antitumour activity of gemcitabine monotherapy [75] and gemcitabine plus docetaxel [88,89] in patients with uterine leiomyosarcoma was also indicated by the non-RCT evidence. The results observed from the non-randomised evidence should be interpreted in light of the inherent limitations associated with this study design. RCTs involve randomisation which minimises the selection bias and confounding, and are therefore the most rigorous way of determining comparative efficacy.

Despite the systematic approach employed in this review, it was limited by the identification of only a small number of RCTs and the lack of comparability in terms of sample size, study design, and patient populations across both the RCTs and non-randomised studies. The patient population included in the RCT by van Oosterom and colleagues varied from those recruited in other RCTs [29]. The study by van Oosterom and colleagues included a mixed patient population of both first-line and second-line patients, with limited subgroup data for the patients treated in the second-line setting [29]. Most importantly, the RCT evidence was restricted by the fact that there was a lack of head-to-head trials of active agents. Due to the paucity of evidence, indirect and mixed treatment comparison of the included interventions were also not possible as no studies evaluating a common intervention were identified, except the two placebo-controlled trials, wherein an indirect analysis was not feasible due to incompatibility of the data (lack of comparability of the study designs and patient population in these studies) [18,19,28]. Although, the review also included 52 prospective non-randomised studies, these studies were small in terms of sample size with majority of studies including less than 50 patients. For several treatments, only single studies were available. When there were multiple studies evaluating a single intervention, variability was often observed in the efficacy and safety results, primarily attributed to differences in patient characteristics and assessment criteria used to evaluate efficacy measures.

With respect to the inclusion criteria of the review, this study was limited to trials evaluating adult patients with sub-types of STS (excluding GIST, Kaposi sarcoma, and Ewing’s family of tumours), who had received prior anthracycline and/or ifosfamide therapy for advanced disease. Based on the inclusion criteria of the review, key RCTs including Maki 2007 (gemcitabine vs. gemcitabine plus docetaxel) and Verweij 2000 (docetaxel vs. doxorubicin) and single arm studies including Sleijfer 2009 (pazopanib) and Bay 2006 (gemcitabine plus docetaxel) were excluded as these publications did not provide information fulfilling the inclusion criteria of the review [92-95]. The study by Maki and colleagues included a mixed population of patients treated with zero to three prior chemotherapy regimens with no data specifically reported for patients receiving ≥1 chemotherapy regimen; in addition, the type of prior chemotherapy was unclear [92]. The RCT by Verweij and colleagues was excluded from the review as the study included patients with GIST [93]. In the study by Sleijfer and colleagues, the type of prior therapy was not reported [94], while in the study by Bay and colleagues no sub-group data for patients receiving first-line and later-lines therapies was provided [95]. Additionally, in the study by Bay and colleagues nearly 69% of patients were treated within the adjuvant setting [95]. A further RCT (TAXOGEM study) investigating gemcitabine vs. gemcitabine plus docetaxel [27,31], whilst identified in our review had not reported efficacy data at the cut-off date for the literature search and thus, while has done subsequently [96], the findings do not contribute to our conclusions.

### Clinical perspective

The primary aim of second and later line treatment of patients with advanced/metastatic soft tissue sarcoma is to delay disease progression and maintain quality of life for as long as possible. The use of an anti-tumour treatment rather than best supportive care should be extensively discussed with the patient and their caregivers. Until now, there has been no standard of care after failure of or intolerance to doxorubicin and/or ifosfamide. An adjusted indirect comparison would be the most appropriate way to compare results of RCTs, but in this case since none of the RCTs had common arms to enable a formal indirect comparison, close attention should be paid to the findings of the individual trials.

In only one trial did the chemotherapy regimen improve overall survival (the combination of gemcitabine plus dacarbazine over dacarbazine alone; Table 2) [20,21]. This trial was not designed to formally demonstrate an overall survival advantage, and therefore, this finding needs to be confirmed by an appropriately designed Phase III trial. Moreover, the sample size of the trial was limited (59 patients received the combination) and there is no other published study investigating this original combination [20,21]. The full results of the TAXOGEM study [96] are not included in our review for reasons explained earlier but demonstrate the activity of gemcitabine plus docetaxel in patients with leiomyosarcomas and may explain the frequent usage of this combination in clinical practice, especially in those with leiomyosarcomas at uterine sites.

The PALETTE trial has formally demonstrated the benefit of treating patients with anti-angiogenic agent over placebo in terms of PFS in a Phase III setting (Table 2) [18,19]. This constitutes a major breakthrough in sarcoma management. However, possibly due to the high usage rate of salvage treatment after progression, this improvement in PFS did not translate into an OS advantage (Table 2).

The every 3 week (q3w) schedule of trabectedin was associated with improvement of PFS, but because of the planned crossover, there was no advantage in term of OS over the weekly schedule (Table 2) [22-26]. Moreover, the weekly schedule may be less convenient than the every 3 week schedule. It should be noted that trabectedin is not currently approved for use in sarcoma in all countries.

Because quality of life and toxicity concerns are of key importance in this setting, the consideration of tolerability and discontinuation rates is as important as efficacy. The traditional cytotoxic drugs commonly induce haematological toxicities whereas grade 3/4 toxicities seen with pazopanib included fatigue, elevated liver enzymes, and hypertension (Table 3). The safety profiles of both approaches (chemotherapy versus pazopanib) appear to be distinct; this is of particular relevance when discussing the toxicity/benefit ratio with patients. Table 4 suggests that discontinuations due to AEs may be more frequent with pazopanib, possibly because oncologists are less familiar with managing the side effects associated with this agent unlike the classical cytotoxic haemotological toxicities, which have been known for years. Discontinuations could also be related to the fact that pazopanib is given continuously unlike cytotoxic therapy, allowing less opportunity for resolution of toxicities.

This review demonstrates that non-randomised trials provide limited information (Table 6). Randomised studies are preferred when designing new trials. The safety profiles of chemotherapy agents versus pazopanib are clearly different, so additional data including compliance, quality of life and cost are needed to fully understand the extent of the differences between chemotherapy and targeted agents.

## Conclusions

Based on this review, the following regimens have demonstrated a PFS advantage: pazopanib over placebo, trabectedin 3-weekly over weekly schedule, and the combination of gemcitabine plus dacarbazine over dacarbazine alone. Consequently, the choice of second- and later-line treatment for advanced STS should consider these interventions. The efficacy/toxicity ratio of therapies which have limited Phase II evidence should be further evaluated in phase III trials based on formal statistical assumptions, and should include parameters such as median overall survival and quality of life.

## Abbreviations

AE: Adverse event; ALT: Alanine aminotransferase; AST: Aspartate aminotransferase; CR: Complete response; DOR: Duration of response; GIST: Gastrointestinal stromal tumour; ORR: Overall response rate; OS: Overall survival; PD: Progressive disease; PFS: Progression-Free survival; PR: Partial response; PRISMA: Preferred reporting items for systematic reviews and meta-analyses; RCTS: Randomised controlled trials; SD: Stable disease; STS: Soft tissue sarcoma; TTP: Time to progression; TTR: Time to response.

## Competing interests

SS and ST are employees of HERON Health Pvt. Ltd., which was commissioned by GSK to undertake the research for this review. SM and SP are employees of GSK. NP declares no competing interests.

## Authors' contributions

SS: participated in the study design, data extraction, article selection, coordination, and manuscript preparation; ST: participated in the article selection, data extraction, and manuscript preparation; SM: participated in study design and coordination and helped to draft the manuscript; SP: conceived of the study, and participated in its design and coordination, and helped to draft the manuscript. NP: helped to draft the manuscript. All authors read and approved the final manuscript.

## Pre-publication history

The pre-publication history for this paper can be accessed here:

http://www.biomedcentral.com/1471-2407/13/385/prepub

## Supplementary Material

Additional file 1 **PRISMA checklist.** This file includes the PRISMA checklist. Click here for file

Additional file 2 **Search strategy for Embase^®^ and MEDLINE^®^.** The file describes the search strategy employed for searching electronic databases Embase^®^ and Medline^®^. Click here for file

Additional file 3 **Quality assessment of RCTs.** The file describes the quality assessment of RCTs undertaken on the following parameters: Randomisation: was randomisation carried out appropriately? Allocation concealment: Was the concealment of treatment allocation adequate? Baseline comparability: Were the groups similar at the outset of the study in terms of prognostic factors, for example, severity of disease? Blinding: Were the care providers, participants and outcome assessors blind to treatment allocation? Follow-up: Were there any unexpected imbalances in drop-outs between groups? Selective reporting: Is there any evidence to suggest that the authors measured more outcomes than they reported? Analysis: Did the analysis include an intention-to-treat analysis? If so, was this appropriate and were appropriate methods used to account for missing data? Other source of bias: Were there any other sources of bias? Click here for file

Additional file 4 **Key Differences Between Regulatory and Academic Analyses of PALETTE Study.** This file highlights the similarities and differences between the two different analyses (regulatory and academic) performed on the PALETTE data. Click here for file

Additional file 5 **Detailed summary of prospective non-randomised studies with sample size more than 10.** This file describes the details of prospective non-randomised studies with sample size more than 10 with respect to study design, sample size, median duration of follow-up, prior therapy, ECOG performance status, and STS sub-type. Click here for file

Additional file 6 **List of prospective small-size studies (sample size <10) and retrospective studies.** This file describes the list of prospective small-size studies (sample size <10) and retrospective studies included in the review. Click here for file

Additional file 7 **Summary of grade 3 or grade 4 specific adverse events observed across non-randomised trials.** This file describes the grade 3/4 AEs observed across non-randomised controlled trials. Click here for file
