# Supplementary material for: Efficacy and safety of pharmacological interventions in second- or later-line treatment of patients with advanced soft tissue sarcoma: a systematic review
Source: BMC Cancer. 2013 Aug 13;13:385. doi: 10.1186/1471-2407-13-385 (PMC3765173; doi:10.1186/1471-2407-13-385)
Supplement: Additional file 2 — Search strategy for Embase® and MEDLINE®. The file describes the search strategy employed for searching electronic databases Embase® and Medline®. [file 1471-2407-13-385-S2.doc]

Additional file 2 - Search strategy for Embase® and MEDLINE®

| S.No | Search history |
| --- | --- |
| #1 | 'clinical trial'/exp |
| #2 | 'randomization'/de |
| #3 | 'controlled study'/de |
| #4 | 'comparative study'/de |
| #5 | 'single blind procedure'/de |
| #6 | 'double blind procedure'/de |
| #7 | 'crossover procedure'/de |
| #8 | 'placebo'/de |
| #9 | 'clinical trial' OR 'clinical trials' |
| #10 | 'controlled clinical trial' OR 'controlled clinical trials' |
| #11 | 'randomised controlled trial' OR 'randomized controlled trial' OR 'randomised controlled trials' OR 'randomized controlled trials' |
| #12 | 'randomisation' OR 'randomization' |
| #13 | rct |
| #14 | 'random allocation' |
| #15 | 'randomly allocated' |
| #16 | 'allocated randomly' |
| #17 | allocated NEAR/2 random |
| #18 | (single OR double OR triple OR treble) NEAR/1 (blind* OR mask*) |
| #19 | placebo* |
| #20 | 'prospective study'/de |
| #21 | #1 OR #2 OR #3 OR #4 OR #5 OR #6 OR #7 OR #8 OR #9 OR #10 OR #11 OR #12 OR #13 OR #14 OR #15 OR #16 OR #17 OR #18 OR #19 OR #20 |
| #22 | 'case study'/de |
| #23 | 'case report' |
| #24 | 'abstract report'/de |
| #25 | 'letter'/de |
| #26 | #22 OR #23 OR #24 OR #25 |
| #27 | #21 NOT #26 |
| #28 | 'cohort analysis'/exp |
| #29 | 'longitudinal study'/exp |
| #30 | 'prospective study'/exp |
| #31 | 'follow up'/exp |
| #32 | 'major clinical study'/exp |
| #33 | 'clinical trial'/exp |
| #34 | 'clinical article'/exp |
| #35 | 'intervention study'/exp |
| #36 | 'survival'/exp |
| #37 | cohort*:ab,ti |
| #38 | (('follow up' OR followup) NEXT/1 (study OR studies)):ab,ti |
| #39 | (clinical NEXT/1 trial*):ab,ti |
| #40 | 'retrospective study'/exp |
| #41 | 'case control study'/exp |
| #42 | (case* NEXT/1 control*):ab,ti |
| #43 | #28 OR #29 OR #30 OR #31 OR #32 OR #33 OR #34 OR #35 OR #36 OR #37 OR #38 OR #39 OR #40 OR #41 OR #42 |
| #44 | 'soft tissue sarcoma'/de |
| #45 | 'soft tissue sarcoma':ab,ti OR 'soft tissue sarcomas':ab,ti OR 'soft tissue cancer':ab,ti OR 'soft part sarcoma':ab,ti OR 'soft part sarcomas':ab,ti OR sts:ab,ti |
| #46 | ('soft tissue' NEAR/2 (tumour* OR tumor*)):ab,ti |
| #47 | 'fibrosarcoma'/de OR fibrosarcom*:ab,ti OR (fibrous NEAR/1 (tumour* OR tumor*)):ab,ti |
| #48 | 'myxosarcoma'/de OR myxofibrosarcom*:ab,ti OR myxosarcom*:ab,ti |
| #49 | 'epithelioid sarcoma'/de OR 'epithelioid sarcoma':ab,ti OR 'epithelioid sarcomas':ab,ti |
| #50 | 'fibrohistiocytoma'/de OR fibrohistiocytom*:ab,ti |
| #51 | 'leiomyosarcoma'/de OR leiomyosarcom*:ab,ti |
| #52 | 'rhabdomyosarcoma'/de OR rhabdomyosarcom*:ab,ti |
| #53 | 'hemangioendotheliosarcoma'/de OR hemangioendotheliosarcom*:ab,ti |
| #54 | 'angiosarcoma'/de OR angiosarcom*:ab,ti OR hemangiosarcom*:ab,ti |
| #55 | 'synovial sarcoma'/de OR 'synovial sarcoma':ab,ti OR 'synovial sarcomas':ab,ti |
| #56 | 'alveolar soft part sarcoma'/de OR 'alveolar soft part sarcoma':ab,ti OR 'alveolar soft part sarcomas':ab,ti |
| #57 | 'clear cell sarcoma'/de OR 'clear cell sarcoma':ab,ti OR 'clear cell sarcomas':ab,ti |
| #58 | 'desmoplastic small round cell tumor'/de OR ('desmoplastic small round cell' NEAR/1 (tumor* OR tumour*)):ab,ti |
| #59 | 'liposarcoma'/de OR liposarcom*:ab,ti |
| #60 | 'lymphangiosarcoma'/de OR lymphangiosarcom*:ab,ti |
| #61 | 'kaposi sarcoma'/de OR kaposi*:ab,ti |
| #62 | 'glomus tumor'/de OR (glomus NEAR/1 (tumor* OR tumour*)):ab,ti OR glomangiosarcom*:ab,ti |
| #63 | 'hemangiopericytoma'/de OR hemangiopericytom*:ab,ti |
| #64 | 'desmoid tumor'/de OR (desmoid NEAR/1 (tumor* OR tumour*)):ab,ti |
| #65 | 'ewing sarcoma'/de OR ewing*:ab,ti |
| #66 | 'dermatofibrosarcoma protuberans'/de OR dermatofibrosarcom*:ab,ti |
| #67 | 'chondrosarcoma'/de OR chondrosarcom*:ab,ti |
| #68 | 'malignant fibrous histiocytoma'/de OR 'malignant fibrous histiocytoma':ab,ti OR 'malignant fibrous histiocytomas':ab,ti OR 'mfh':ab,ti |
| #69 | 'neurofibrosarcoma'/de OR neurofibosarcom*:ab,ti OR schwannom*:ab,ti |
| #70 | 'mesenchymoma'/de OR mesenchymom*:ab,ti |
| #71 | 'spindle cell sarcoma'/de OR 'spindle cell sarcoma':ab,ti OR 'spindle cell sarcomas':ab,ti |
| #72 | ('spindle cell' NEAR/1 (tumor* OR tumour*)):ab,ti |
| #73 | 'nerve sheath tumor'/exp OR ('nerve sheath' NEAR/1 (tumor* OR tumour*)):ab,ti |
| #74 | 'hemangioendothelioma'/de OR hemangioendotheliom*:ab,ti |
| #75 | fibroblastom*:ab,ti |
| #76 | 'pleomorphic sarcoma':ab,ti OR 'pleomorphic sarcomas':ab,ti OR 'pleomorphic undifferentiated sarcoma':ab,ti OR 'pleomorphic undifferentiated sarcomas':ab,ti |
| #77 | (rhabdoid NEAR/1 (tumor* OR tumour*)):ab,ti |
| #78 | (intimal NEAR/1 sarcoma*):ab,ti |
| #79 | 'perivascular epithelioid cell tumor'/de |
| #80 | ('perivascular epithelioid cell' NEAR/1 (tumor* OR tumour*)):ab,ti |
| #81 | 'pleomorphic adenoma'/de |
| #82 | 'pleomorphic adenoma':ab,ti OR 'pleomorphic adenomas':ab,ti |
| #83 | #44 OR #45 OR #46 OR #47 OR #48 OR #49 OR #50 OR #51 OR #52 OR #53 OR #54 OR #55 OR #56 OR #57 OR #58 OR #59 OR #60 OR #61 OR #62 OR #63 OR #64 OR #65 OR #66 OR #67 OR #68 OR #69 OR #70 OR #71 OR #72 OR #73 OR #74 OR #75 OR #76 OR #77 OR #78 OR #79 OR #80 OR #81 OR #82 |
| #84 | 'brostallicin'/de OR pnu166196:ab,ti OR 'pnu-166196':ab,ti OR 'pnu 166196':ab,ti OR brostallicin:ab,ti OR 'pnu 166196a':ab,ti OR 'pnu166196a':ab,ti |
| #85 | 'carboplatin'/de OR paraplat*:ab,ti OR blastocarb:ab,ti OR carboplat*:ab,ti OR carbosin:ab,ti OR carbosol:ab,ti OR carbotec:ab,ti OR displata:ab,ti OR ercar:ab,ti OR nealorin:ab,ti OR novoplatinum:ab,ti OR platinwas:ab,ti OR ribocarbo:ab,ti OR cbdca:ab,ti OR 'jm 8':ab,ti |
| #86 | 'cetuximab'/de OR imc225:ab,ti OR 'c 225':ab,ti OR c225:ab,ti OR erbitux:ab,ti OR 'imc-c225':ab,ti OR 'imc 225':ab,ti OR 'imc c225':ab,ti OR 'imcc 225':ab,ti OR 'imcc225':ab,ti OR cetuximab:ab,ti |
| #87 | 'cisplatin'/de OR cisplat*:ab,ti OR abiplatin:ab,ti OR platino*:ab,ti OR cddp:ab,ti OR 'cis-platinum':ab,ti OR neoplatin:ab,ti OR 'nk 801':ab,ti OR 'nsc 119875':ab,ti OR plat?mine:ab,ti OR platosin:ab,ti OR randa:ab,ti OR romcis:ab,ti OR 'spi 077':ab,ti OR blastolem:ab,ti OR briplatin:ab,ti OR cisplatyl:ab,ti OR citoplatino:ab,ti OR citosin:ab,ti OR lederplatin:ab,ti OR metaplatin:ab,ti OR placis:ab,ti OR platiblastin:ab,ti OR platinex:ab,ti OR platiran:ab,ti OR platistin OR 'cis ddp':ab,ti OR 'mpi 5010':ab,ti OR mpi5010:ab,ti OR bioc?splatinum:ab,ti OR platidiam:ab,ti |
| #88 | 'cyclophosphamide'/de OR 'b 518':ab,ti OR b518:ab,ti OR carloxan:ab,ti OR clafen:ab,ti OR cycloblastin*:ab,ti OR 'cyclofos amide':ab,ti OR cyclofosfamid*:ab,ti OR cyclophosphamid*:ab,ti OR cyclophosphan*:ab,ti OR cyclostin:ab,ti OR cycloxan:ab,ti OR cyphos:ab,ti OR cytophosphan*:ab,ti OR cytoxan:ab,ti OR 'endocyclo phosphate':ab,ti OR end?xan*:ab,ti OR genoxal:ab,ti OR mitoxan:ab,ti OR neosan:ab,ti OR neosar:ab,ti OR noristan:ab,ti OR 'nsc 26271':ab,ti OR 'nsc 2671':ab,ti OR procytox:ab,ti OR procytoxide:ab,ti OR se?doxan:ab,ti |
| #89 | 'dacarbazine'/de OR peticene:ab,ti OR dacarbazin*:ab,ti OR 'dtic-dome':ab,ti OR dtic:ab,ti OR 'dtic dome':ab,ti OR 'nsc 45388':ab,ti OR nsc45388:ab,ti OR asercit:ab,ti OR dacatic:ab,ti OR detimedac:ab,ti OR fauldetic:ab,ti OR 'imidazole carboxamide':ab,ti OR imidazolecarboxamide:ab,ti OR dic:ab,ti OR 'wr-139007':ab,ti |
| #90 | 'deforolimus'/de OR ap23573:ab,ti OR 'ap 23573':ab,ti OR mk8669:ab,ti OR 'mk 8669':ab,ti OR ridaforolimus:ab,ti OR deforolimus:ab,ti |
| #91 | 'docetaxel'/de OR docetaxel:ab,ti OR taxotere:ab,ti OR 'rp 56976':ab,ti OR rp56976:ab,ti |
| #92 | 'doxorubicin'/de OR doxorubicin*:ab,ti OR adriablastin:ab,ti OR adriablastin*:ab,ti OR adriacin:ab,ti OR adriamicin*:ab,ti OR adriblastin*:ab,ti OR caelyx:ab,ti OR doxil:ab,ti OR 'fi 106':ab,ti OR fi106:ab,ti OR myocet:ab,ti OR 'nsc 123127':ab,ti OR nsc123127:ab,ti OR rastocin:ab,ti OR resmycin:ab,ti OR 'rp 25253':ab,ti OR rp25253:ab,ti OR rubex:ab,ti OR sarcodoxome:ab,ti OR 'tlc d 99':ab,ti OR adriamycin:ab,ti OR 'dox-sl':ab,ti OR evacet:ab,ti OR lipodox:ab,ti OR doxilen:ab,ti OR 'tlc d-99':ab,ti |
| #93 | 'epirubicin'/de OR pharmorubicin*:ab,ti OR epirubicin:ab,ti OR ellence:ab,ti OR epidoxorubicin:ab,ti OR pidorubicin:ab,ti OR epiadriamycin:ab,ti OR epidx:ab,ti OR farmorubicin*:ab,ti OR 'imi 28':ab,ti OR imi28:ab,ti OR 'imi-28':ab,ti OR 'nsc 256942':ab,ti OR nsc256942:ab,ti OR 'epi dx':ab,ti OR epi:ab,ti OR 'epi-adr':ab,ti |
| #94 | 'erlotinib'/de OR cp358774:ab,ti OR tarceva:ab,ti OR 'cp 358774':ab,ti OR erlotinib:ab,ti OR 'nsc 718781':ab,ti OR nsc718781:ab,ti OR 'osi 774':ab,ti OR osi774:ab,ti OR 'r 1415':ab,ti OR r1415:ab,ti |
| #95 | 'etoposide'/de OR etoposide:ab,ti OR toposar:ab,ti OR vepesid:ab,ti OR lastet:ab,ti OR epeg:ab,ti OR 'vp 16':ab,ti OR 'vp 16 213':ab,ti |
| #96 | 'everolimus'/de OR everolimus:ab,ti OR afinitor:ab,ti OR certican:ab,ti OR 'nvp-rad-001':ab,ti OR 'rad-001':ab,ti OR 'rad 001a':ab,ti OR rad001:ab,ti OR rad001a:ab,ti OR 'sdz rad':ab,ti OR xience:ab,ti |
| #97 | 'gefitinib'/de OR gefitinib:ab,ti OR iressa:ab,ti OR 'zd 1839':ab,ti OR zd1839:ab,ti |
| #98 | 'gemcitabine'/de OR gemzar:ab,ti OR gemcitabine:ab,ti OR 'ly 188011':ab,ti OR ly188011:ab,ti OR 'ly-188011':ab,ti OR dfdcyd:ab,ti OR dfdc:ab,ti |
| #99 | 'ifosfamide'/de OR ifosfamid*:ab,ti OR 'asta 2 4942':ab,ti OR 'asta 24942':ab,ti OR 'asta 4942':ab,ti OR 'asta z 4942':ab,ti OR 'asta z-4942':ab,ti OR 'asta z4942':ab,ti OR asta24942:ab,ti OR asta4942:ab,ti OR cyfos:ab,ti OR holoxan*:ab,ti OR ifex:ab,ti OR ifomid*:ab,ti OR iphosphamid*:ab,ti OR isocyclophosphamide:ab,ti OR isoendoxan:ab,ti OR 'iso-endoxan':ab,ti OR isofosfamid*:ab,ti OR isophosphamid*:ab,ti OR mitoxana:ab,ti OR 'mjf 9325':ab,ti OR mjf9325:ab,ti OR naxamide:ab,ti OR 'nsc 109724':ab,ti OR nsc109724:ab,ti OR 'z 4942':ab,ti OR z4942:ab,ti OR 'ifo-cell':ab,ti OR ifolem:ab,ti OR ifoxan:ab,ti OR tronoxal:ab,ti OR 'nsc-109,724':ab,ti OR 'nsc 109,724':ab,ti OR 'nsc109,724':ab,ti |
| #100 | 'methotrexate'/de OR methotrexat*:ab,ti OR mtx:ab,ti OR 'a methopterine':ab,ti OR abitrexate:ab,ti OR amethopterin*:ab,ti OR ametopterin*:ab,ti OR antifolan:ab,ti OR 'cl 14377':ab,ti OR cl14377:ab,ti OR e?thexat*:ab,ti OR emtrexate:ab,ti OR farmitrexat*:ab,ti OR folex:ab,ti OR ledertrexate:ab,ti OR methoblastin:ab,ti OR methohexate:ab,ti OR methotrate:ab,ti OR methoxtrexat*:ab,ti OR methrotrexat*:ab,ti OR methylaminopterin*:ab,ti OR metothrexat*:ab,ti OR metotrexat*:ab,ti OR mexate:ab,ti OR 'mpi 5004':ab,ti OR mpi5004:ab,ti OR novatrex:ab,ti OR 'nsc 740':ab,ti OR nsc740:ab,ti OR rheumatrex:ab,ti OR trexall:ab,ti OR brimexate:ab,ti OR emtexate:ab,ti OR fauldexato:ab,ti OR lantarel:ab,ti OR lumexon:ab,ti OR maxtrex:ab,ti OR medsatrexate:ab,ti OR metex:ab,ti OR metrotex:ab,ti OR texate:ab,ti OR tremetex:ab,ti OR trexeron:ab,ti OR trixilem:ab,ti OR 'cl-14377':ab,ti OR 'wr-19039':ab,ti |
| #101 | 'paclitaxel'/de OR paclitaxel:ab,ti OR taxol:ab,ti OR anzatax:ab,ti OR asotax:ab,ti OR bristaxol:ab,ti OR praxel:ab,ti OR 'taxol konzentrat':ab,ti |
| #102 | 'pazopanib'/de OR armala:ab,ti OR pazopanib:ab,ti OR gw786034*:ab,ti OR (gw NEXT/1 786034*):ab,ti OR (sb NEXT/1 710468*):ab,ti OR sb710468*:ab,ti OR votrient:ab,ti |
| #103 | 'sorafenib'/de OR 'bay 43-9006':ab,ti OR 'bay 439006':ab,ti OR 'bay43-9006':ab,ti OR bay439006:ab,ti OR nexavar:ab,ti OR sorafenib:ab,ti OR 'bay 54-9085':ab,ti |
| #104 | 'sunitinib'/de OR sunitinib:ab,ti OR sutent:ab,ti OR 'pha 2909040ad':ab,ti OR 'pha2909040ad':ab,ti OR 'su 010398':ab,ti OR 'su 011248':ab,ti OR 'su 10398':ab,ti OR su10398:ab,ti OR 'su 11248':ab,ti OR su010398:ab,ti OR 'su011248':ab,ti OR su11248:ab,ti |
| #105 | 'tamoxifen'/de OR tamoxifen*:ab,ti OR nolvadex:ab,ti OR novaldex:ab,ti OR 'apo-tamox':ab,ti OR clonoxifen:ab,ti OR dignotamoxi:ab,ti OR ebefen:ab,ti OR emblon:ab,ti OR estroxyn:ab,ti OR fentamox:ab,ti OR genox:ab,ti OR jenoxifen:ab,ti OR kessar:ab,ti OR ledertam:ab,ti OR lesporene:ab,ti OR nolgen:ab,ti OR noltam:ab,ti OR nourytam:ab,ti OR novofen:ab,ti OR oestrifen:ab,ti OR oncotam:ab,ti OR soltamox:ab,ti OR tamax*:ab,ti OR tam?fen:ab,ti OR tamizam:ab,ti OR tamoxasta:ab,ti OR zemide:ab,ti OR 'nsc 180973':ab,ti OR tamoplac:ab,ti OR 'ici 46,474':ab,ti OR 'ici-46474':ab,ti OR istubal:ab,ti OR valodex:ab,ti |
| #106 | 'temsirolimus'/de OR temsirolimus:ab,ti OR 'cci-779':ab,ti OR 'cell-cycle-inhibitor-779':ab,ti OR 'nsc 683864':ab,ti OR nsc683864:ab,ti OR torisel:ab,ti |
| #107 | 'trabectedin'/de OR et743:ab,ti OR ecteinascidin:ab,ti OR 'et 743':ab,ti OR yondelis:ab,ti OR trabectedin:ab,ti |
| #108 | 'vinblastine'/de OR vinblastin*:ab,ti OR vincaleucoblastine:ab,ti OR velban:ab,ti OR velsar:ab,ti OR vlb:ab,ti OR velbe:ab,ti |
| #109 | 'vincristine'/de OR vincristin*:ab,ti OR 'l 37231':ab,ti OR l37231:ab,ti OR 'vin cristine':ab,ti OR vincrisul:ab,ti OR oncovin:ab,ti |
| #110 | #84 OR #85 OR #86 OR #87 OR #88 OR #89 OR #90 OR #91 OR #92 OR #93 OR #94 OR #95 OR #96 OR #97 OR #98 OR #99 OR #100 OR #101 OR #102 OR #103 OR #104 OR #105 OR #106 OR #107 OR #108 OR #109 |
| #111 | #27 AND #83 AND #110 |
| #112 | #43 AND #83 AND #110 |
| #113 | #111 OR #112 |
