# Supplementary material for: Efficacy and safety of pharmacological interventions in second- or later-line treatment of patients with advanced soft tissue sarcoma: a systematic review
Source: BMC Cancer. 2013 Aug 13;13:385. doi: 10.1186/1471-2407-13-385 (PMC3765173; doi:10.1186/1471-2407-13-385)
Supplement: Additional file 3 — Quality assessment of RCTs. The file describes the quality assessment of RCTs undertaken on the following parameters: Randomisation: was randomisation carried out appropriately? Allocation concealment: Was the concealment of treatment allocation adequate? Baseline comparability: Were the groups similar at the outset of the study in terms of prognostic factors, for example, severity of disease? Blinding: Were the care providers, participants and outcome assessors blind to treatment allocation? Follow-up: Were there any unexpected imbalances in drop-outs between groups? Selective reporting: Is there any evidence to suggest that the authors measured more outcomes than they reported? Analysis: Did the analysis include an intention-to-treat analysis? If so, was this appropriate and were appropriate methods used to account for missing data? Other source of bias: Were there any other sources of bias? [file 1471-2407-13-385-S3.doc]

Additional file 3 - Quality assessment of RCTs

Critical appraisal of included studies conducted using a comprehensive assessment criteria based on the recommendations in the NICE manufacturer’s template and the Cochrane’s critical appraisal tool is summarised in the table below

| Study | PALETTE study 2011 | Demetri 2009 | GEIS study | Pautier 2009 | Pacey 2009 | van Oosterom 2002 |
| --- | --- | --- | --- | --- | --- | --- |
| Randomisation | Low risk | Not clear | Not clear | Not clear | Not clear | Not clear |
| Allocation concealment | Low risk | Not clear | Not clear | Not clear | Low risk | Low risk |
| Baseline comparability | Low risk | Low risk | Low risk | Not clear | Not clear | Low risk |
| Blinding | Low risk | Low risk | Not clear | Not clear | Not clear | Not clear |
| Follow-up | Low risk | Low risk | Low risk | Not clear | Not clear | Not clear |
| Selective reporting | Low risk | Not clear | Not clear | Not clear | Not clear | Not clear |
| Analysis | Low risk | Low risk | Low risk | Not clear | Low risk | Low risk |
| Other source of bias | Low risk | Not clear | Not clear | Not clear | Not clear | Not clear |

Randomisation: was randomisation carried out appropriately? Allocation concealment: Was the concealment of treatment allocation adequate? Baseline comparability: Were the groups similar at the outset of the study in terms of prognostic factors, for example, severity of disease? Blinding: Were the care providers, participants and outcome assessors blind to treatment allocation? Follow-up: Were there any unexpected imbalances in drop-outs between groups? Selective reporting: Is there any evidence to suggest that the authors measured more outcomes than they reported? Analysis: Did the analysis include an intention-to-treat analysis? If so, was this appropriate and were appropriate methods used to account for missing data? Other source of bias: Were there any other sources of bias?
