# Supplementary material for: Efficacy and safety of pharmacological interventions in second- or later-line treatment of patients with advanced soft tissue sarcoma: a systematic review
Source: BMC Cancer. 2013 Aug 13;13:385. doi: 10.1186/1471-2407-13-385 (PMC3765173; doi:10.1186/1471-2407-13-385)
Supplement: Additional file 4 — Key Differences Between Regulatory and Academic Analyses of PALETTE Study. This file highlights the similarities and differences between the two different analyses (regulatory and academic) performed on the PALETTE data. [file 1471-2407-13-385-S4.docx]

Additional File 4 – Key Differences Between Regulatory and Academic Analyses of PALETTE Study

| **Variable** | **Regulatory Analysis from Clinical Study Report (used in this publication)** | **Academic Analysis (Van Der Graaf 2012, Van Der Graaf 2011, Coens 2012)** |
| --- | --- | --- |
| **Median Progression Free Survival by Independent Review** | **1.6 m (PLB) vs 4.6m (PAZ); HR=0.35 (0.26, 0.48); p<0.001** | **1.6 m (PLB) vs 4.6m (PAZ); HR=0.31 (0.24, 0.40); p<0.0001** |
| **PFS by Subtype**  **(ITT Population)** | **Leiomyosarcoma HR=0.37 (0.23, 0.60); p<0.001**  **Synovial HR=0.43 (0.19, 0.98); p=0.005**  **Others HR=0.39 (0.25, 0.60) p<0.001** | **Leiomyosarcoma HR=0.31 (0.20, 0.47); p<0.0001**  **Synovial HR^ᵻ^=0.19 (0.07, 0.43); p=0.0002**  **Others HR=0.36 (0.25, 0.52) p<0.0001** |
| **Median Overall Survival** | **10.7m (PLB) vs 12.6m (PAZ); HR= 0.87 (0.67, 1.13); p=0.256** | **10.7m (PLB) vs 12.5m (PAZ); HR= 0.86 (0.67, 1.11); p=0.2514** |
| **Confirmed Response by Independent Review** | **Placebo:CR–0%; PR–0%; SD–27%; PD–62%**  **Pazopanib:CR–0%; PR–4%; SD–54%; PD-27%** | **Placebo:CR–0%; PR–0%; SD–38%; PD–57%**  **Pazopanib:CR–0%; PR–6%; SD–67%; PD-23%** |
| **AEs of Interest on Pazopanib (%)**  **Test**   \| **Fatigue** \| \| --- \| \| **Diarrhea** \| \| **Nausea** \| \| **Weight Loss** \| \| **Hypertension** \| \| **Decreased Appetite** \| \| **Hair Colour Changes** \| \| **Vomiting** \| \| **Dysgeusia** \| \| **Exfoliative Rash** \| \| **Myocardial Dysfunc.** \| \| **VTE** \| \| **Pneumothorax** \| \| **ALT Increase** \| \| **AST Increase** \| \| **Total Bilirubin** \| \|  \| | \| **Placebo** \| \| \| **Pazopanib** \| \| \| \| --- \| --- \| --- \| --- \| --- \| --- \| \| **All** \| **Gr3** \| **Gr4** \| **All** \| **Gr3** \| **Gr4** \| \| **48** \| **4** \| **<1** \| **65** \| **13** \| **<1** \| \| **15** \| **<1** \| **0** \| **59** \| **5** \| **0** \| \| **22** \| **2** \| **0** \| **56** \| **3** \| **0** \| \| **15** \| **0** \| **0** \| **48** \| **4** \| **0** \| \| **6** \| **0** \| **0** \| **42** \| **7** \| **0** \| \| **19** \| **0** \| **0** \| **40** \| **6** \| **0** \| \| **2** \| **0** \| **0** \| **39** \| **0** \| **0** \| \| **11** \| **<1** \| **0** \| **33** \| **3** \| **0** \| \| **3** \| **0** \| **0** \| **28** \| **0** \| **0** \| \| **9** \| **0** \| **0** \| **18** \| **<1** \| **0** \| \| **5** \| **0** \| **0** \| **9** \| **1** \| **<1** \| \| **2** \| **<1** \| **2** \| **5** \| **2** \| **<1** \| \| **0** \| **0** \| **0** \| **3** \| **<1** \| **<1** \| \| **18** \| **3** \| \| **46** \| **10** \| \| \| **22** \| **2** \| \| **51** \| **8** \| \| \| **7** \| **2** \| \| **29** \| **1** \| \| \|  \|  \| \|  \|  \| \| | \| **Placebo** \| \| \| **Pazopanib** \| \| \| \| --- \| --- \| --- \| --- \| --- \| --- \| \| **All** \| **Gr 3** \| **Gr 4** \| **All** \| **Gr 3** \| **Gr 4** \| \| **49** \| **5** \| **1** \| **65** \| **13** \| **<1** \| \| **16** \| **1** \| **0** \| **58** \| **5** \| **0** \| \| **28** \| **2** \| **0** \| **54** \| **3** \| **0** \| \| **20** \| **0** \| **0** \| **48** \| **0** \| **0** \| \| **7** \| **3** \| **0** \| **41** \| **7** \| **0** \| \| **20** \| **0** \| **0** \| **40** \| **6** \| **0** \| \| **2** \| **0** \| **0** \| **38** \| **0** \| **0** \| \| **11** \| **1** \| **0** \| **33** \| **3** \| **0** \| \| **4** \| **0** \| **0** \| **27** \| **0** \| **0** \| \| **11** \| **0** \| **0** \| **18** \| **<1** \| **0** \| \| **5** \| **0** \| **0** \| **9** \| **1** \| **<1** \| \| **2** \| **<1** \| **1** \| **5** \| **2** \| **<1** \| \| **0** \| **0** \| **0** \| **3** \| **0** \| **<1** \| \|  \| **3** \| \|  \| **10** \| \| \|  \| **2** \| \|  \| **8** \| \| \|  \| **2** \| \|  \| **2** \| \| \|  \|  \| \|  \|  \| \| |
| **AE as Primary Reason for Study Drug Discontinuation** | **Placebo 2%; Pazopanib 17%** | **Placebo 1%; Pazopanib 14%** |
| **Median Duration of Treatment** | **Placebo 8.1 weeks (range 1.1-101.9w)**  **Pazopanib 19.4 weeks (range 0.3-102.9w)** | **Placebo 8.1 weeks (range 1-52w)**  **Pazopanib 16.4 weeks (range 0-79w)** |
| **Quality of Life – EORTC QLQ-C30 Questionnaire, Global Health Status (Minimally Important Difference = 5 to 10)** | **Difference Between Arms* (CI) at**  **Baseline: 1.5**  **Week 4: -2.9 (-7.6, 1.7)**  **Week 8: -2.1 (-7.6, 3.4)**  **Week 12: -0.3 (-6.6, 6.0)** | **Difference Between Arms* (CI) at**  **Baseline: 1.4 (-3.7, 6.4)**  **Week 4: -3.8 (-9.0, 1.3)**  **Week 8: -2.3 (-8.3, 3.7)**  **Week 12: -1.6 (-8.4, 5.1)** |
| **Patient Characteristics**   \| **Histology** \| \| --- \| \| LMS \| \| Synovial \| \| Other \| \| **Median Time Since Diagnosis (m)** \| \| **Median Time Since Last Progression (m)** \| \| **Prior Lines of Tx** \| \| 0-1 prior line \| \| 2+ prior lines \| \| **WHO PS^^^** \| \| 0 \| \| 1 \| \| **Prior systemic anti-cancer therapy** \| \| Doxorubicin \| \| Ifosfamide \| \| Docetaxel \| \| Gemcitabine \| \| Trabectedin \| \| mTOR Inhibitors \| \| Other \| \|  \| | \| **Placebo** \| **Pazopanib** \| \| --- \| --- \| \|  \|  \| \| **40%** \| **44%** \| \| **11%** \| **10%** \| \| **50%** \| **46%** \| \| **27.0** \| **26.6** \| \| **0.6** \| **0.7** \| \|  \|  \| \| **42%** \| **45%** \| \| **58%** \| **55%** \| \|  \|  \| \| **49%** \| **48%** \| \| **51%** \| **52%** \| \| **Placeholder part 2** \|  \| \| **98%** \| **98%** \| \| **76%** \| **67%** \| \| **28%** \| **28%** \| \| **34%** \| **35%** \| \| **18%** \| **15%** \| \| **2%** \| **4%** \| \| **43%** \| **43%** \| \|  \|  \| | \| **Placebo** \| **Pazopanib** \| \| --- \| --- \| \|  \|  \| \| **41%** \| **47%** \| \| **11%** \| **12%** \| \| **48%** \| **41%** \| \|  \|  \| \|  \|  \| \|  \|  \| \| **42%** \| **45%** \| \| **58%** \| **55%** \| \|  \|  \| \| **46%** \| **46%** \| \| **54%** \| **54%** \| \| **Placeholder part 2** \|  \| \| **98%** \| **99%** \| \| **76%** \| **67%** \| \| **29%** \| **28%** \| \| **34%** \| **35%** \| \| **18%** \| **15%** \| \| **5%** \| **7%** \| \|  \|  \| \|  \|  \| |
