# Supplementary material for: Efficacy and safety of pharmacological interventions in second- or later-line treatment of patients with advanced soft tissue sarcoma: a systematic review
Source: BMC Cancer. 2013 Aug 13;13:385. doi: 10.1186/1471-2407-13-385 (PMC3765173; doi:10.1186/1471-2407-13-385)
Supplement: Additional file 5 — Detailed summary of prospective non-randomised studies with sample size more than 10. This file describes the details of prospective non-randomised studies with sample size more than 10 with respect to study design, sample size, median duration of follow-up, prior therapy, ECOG performance status, and STS sub-type. [file 1471-2407-13-385-S5.doc]

Additional file 5 - Detailed summary of prospective non-randomised studies with sample size more than 10

| Intervention | Study | Study design | N | Median (range) age, years | Males (%) | Median duration of follow-up (weeks) | Prior therapy received | ECOG performance status, n (%) | STS subtypes (%) |
| --- | --- | --- | --- | --- | --- | --- | --- | --- | --- |
| Brostallicin | Leahy 2007 | Phase II, SA | 43 | 51 (25 - 75) | 44.2% | - | Doxorubicin and/or ifosfamide: 100% | PS 0: 23 (53.5); PS 1: 20 (46.5) | Angiosarcoma: 2.3%; Fibrosarcoma: 2.3%; Leiomyosarcoma: 20.9%; Liposarcoma: 14.0%; MFH: 4.7%; Rhabdomyosarcoma: 2.3%; Synovial sarcoma: 20.9%; Undifferentiated sarcoma and sarcoma (NOS): 25.6%; Other histotypes: 7.0% |
| Cisplatin | Thigpen 1986 | Phase II, SA | 20 | (48 - 70) | - | - | Anthracycline: 100% | PS 0, 1, 2: 18 (94.7); PS 3: 1 (5.3) | Leiomyosarcoma: 100% |
| Cyclophosphamide | Bramwell 1993 | Phase II, SA | 18 | - | - | - | Ifosfamide: 100% | - | STS (non-specified): 100% |
| Dacarbazine | Buesa 1991 | Phase II, SA | 47 | 51 (18 - 73) | 52.3% | - | Anthracycline: 95.5%; ifosfamide: 86%^ | PS 0: 15 (34.1); PS 1: 22 (50.0); PS 2: 7 (15.9) | Fibrosarcoma: 9.1%; Leiomyosarcoma: 27.3%; Liposarcoma: 6.8%; MFH: 13.6%; Synovial sarcoma: 15.9%; Neurofibrosarcoma: 6.8%; Miscellaneous: 20.5% |
| Docetaxel | Kostler, 2001 | Phase II, SA | 25 | - | - | 91.0 weeks (17.3 weeks - 192.4 weeks) | Anthracycline and ifosfamide: 100% | - | Angiosarcoma: 8.0%; Fibrosarcoma: 8.0%; Leiomyosarcoma: 24.0%; Liposarcoma: 8.0%; MFH: 12.0%; Synovial sarcoma: 16.0%; Epitheloid sarcoma: 12.0%; Other: 12.0% |
| Docetaxel | Santoro 1999 | Phase II, SA | 37 | 44 (20 - 64) | 51.4% | - | Anthracycline: 100% | PS 3: 1 (2.7) | Fibrosarcoma: 5.4%; Leiomyosarcoma: 24.3%; Liposarcoma: 5.4%; MFH: 10.8%; Rhabdomyosarcoma: 5.4%; Synovial sarcoma: 21.6%; Schwannoma: 10.8%; Emangiopericytoma: 2.7%; Epithelioid: 2.7%; Unclassified sarcoma: 10.8% |
| Docetaxel | van Hoesel, 1994 | Phase II, SA | 21 | - | - | - | Anthracycline and ifosfamide: 100% | - | STS (non-specified): 100% |
| Doxorubicin | Mouridsen 1987 | Phase II/III, SA | 23 | - | - | - | Anthracycline: 100% | - | STS (non-specified): 100% |
| Etoposide | Crawley 1997 | Phase I/II, SA | 17 | 47 (26 - 71) | 58.8% | - | Anthracycline and/or ifosfamide: 100% | PS 0: 1 (5.9);  PS 1: 12; (70.6); PS 2: 4 (23.5) | Leiomyosarcoma: 52.9%; Liposarcoma: 17.6%; MFH: 5.9%; Synovial sarcoma: 5.9%; MPNST: 11.8%; High grade not classified: 5.9% |
| Etoposide | Dombernowsky 1987 | Phase II, SA | 26 | 47 (17 - 72) | 61.5% | - | Anthracycline: 92.3% | - | STS (non-specified): 100% |
| Gefitinib | Ray-Coquard 2008 | Phase II, SA | 48 | 42 (19 - 66) | 56% | - | Anthracycline and/or ifosfamide: 100% | PS 0: 23; (47.9); PS 1: 20 (41.7); PS 2: 5 (10.4) | Synovial sarcoma: 95.8%; Others: 4.2% |
| Gemcitabine | Ferraresi 2008 | Phase II, SA | 14 | 52 (27 - 77) | 21.4% | - | Anthracycline: 14.3%; ifosfamide: 14.3%; anthracycline and ifosfamide: 64.3%; other: 7.1% | PS 0: 12; (85.7); PS 1: 2 (14.3) | Leiomyosarcoma: 50.0%; MFH: 7.1%; Synovial sarcoma: 7.1%; Malignant schwannoma: 21.4%; Undifferentiated sarcoma: 7.1%; Endometrial stromal cell sarcoma: 7.1% |
| Gemcitabine | Hartmann 2006 | Phase II, SA | 15 | 47 (32 - 72) | 67% | - | Anthracycline: 100%; ifosfamide: 73.3% | PS 0: 11 (73.3); PS 1: 4 (26.7) | Leiomyosarcoma: 20.0%; MFH: 40.0%; Rhabdomyosarcoma: 6.7%; |
| Gemcitabine | Look 2004 | Phase II, SA | 35 | - | - | - | Anthracycline: 54.3%; ifosfamide: 8.6%; anthracycline and ifosfamide: 34.3%; other: 2.9^$^ | - | Leiomyosarcoma: 100% |
| Gemcitabine | Spath-Schwalbe 2000 | Phase II, SA | 18 | 58 (20 - 70) | 55.6% | - | Anthracycline: 88.9%; ifosfamide: 83.3%; anthracycline and/or ifosfamide: 100%; other: 50.0% | - | Leiomyosarcoma: 33.3%; Liposarcoma: 11.1%; MFH: 27.8%; Malignant Schwannoma: 22.2%; clear cell sarcoma: 5.6% |
| Ifosfamide | Antman 1985 | Phase II, SA | 31 | 49.8 (13.0)^$^ | 32.3% | - | Anthracycline: 100% | - | Angiosarcoma: 3.2%; Fibrosarcoma: 3.2%; Leiomyosarcoma: 38.7%; Liposarcoma: 3.2%; MFH: 19.4%; Rhabdomyosarcoma: 3.2%; Epithelioma: 3.2%; neurosarcoma 9.7%;  Mesotheolioma: 12.9%; Spindle sarcoma: 3.2% |
| Ifosfamide | Antman 1989 | Phase II, SA | 94 | - | - | - | All but one patient (who had congestive heart failure and could not receive doxorubicin) had received anthracyclin | - | Angiosarcoma: 2.1%; Fibrosarcoma: 5.3%; Leiomyosarcoma: 28.7%; Liposarcoma: 4.3%; MFH: 7.5%; Rhabdomyosarcoma: 12.8%; Synovial sarcoma: 10.6%; Schwannoma: 6.4%; Mesothelioma: 7.4%; Extraskeletal osteosarcoma: 1.1%; Others: 13.8% |
| Ifosfamide | Babovic 1998 | Unclear, SA | 21 | 50 (20 – 60) | 66. 7% | - | Anthracycline: 100% | PS 0: 4 (19.0); PS 1: 14; (66.7); PS 2: 4 (19.0) | Angiosarcoma: 4.8%; Fibrosarcoma: 4.8%; Leiomyosarcoma: 9.5%; Liposarcoma: 19.0%; MFH: 23.8%; Rhabdomyosarcoma: 4.8%; Synovial sarcoma: 19.0%; Small round cell sarcoma: 4.8%; Malignant schwannoma: 9.5% |
| Ifosfamide | Le Cesne 1995 | Phase II, SA | 40 | 46 (22 – 71) | 47.5% | 73.7 weeks | Anthracycline: 97.5%; ifosfamide: 70.0%; anthracycline and ifosfamide: 65.0%; other: 7.5‡ | PS 0: 12; (30.0); PS 1: 23 (57.5); PS 2: 5 (12.5) | Angiosarcoma: 5.0%; Fibrosarcoma: 12.5%; Leiomyosarcoma: 30.0%; Liposarcoma: 7.5%; MFH: 7.5%; Rhabdomyosarcoma: 5.0%; Synovial sarcoma: 10.0%; Unclassified/undifferentiated sarcoma: 5.0%; neurosarcoma: 17.5% |
| Ifosfamide | Nielsen 2000 | Phase II, SA | 13 | - | - | - | Ifosfamide: 100% | - | STS (non-specified): 100% |
| Ifosfamide | Palumbo 1997 | Phase II, SA | 38 | 48 (21 - 72) | - | - | Anthracycline: 100%; ifosfamide: 73.7% | PS 0: 9^#^ (23.7); PS 1: 22^#^ (57.9); PS 2: 7^#^ (18.4) | Angiosarcoma: 7.9%; Fibrosarcoma: 5.3%; Leiomyosarcoma: 23.7%; Liposarcoma: 18.4%; MFH: 29%; Rhabdomyosarcoma: 5.3%; Synovial sarcoma: 10.5%; |
| Ifosfamide | Patel 1997 | Unclear, SA | 12 | - | - | - | Anthracycline: 100% | - | Leiomyosarcoma: 8.3%; Liposarcoma: 8.3%; MFH: 33.3%; Rhabdomyosarcoma: 0.0%; Synovial sarcoma: 16.7%; Others: 33.3% |
| Ifosfamide | Patel 1997 | Phase II, SA | 32 | - | - | - | Anthracycline: 100% | - | Liposarcoma: 12.5%; MFH: 25%; Rhabdomyosarcoma: 12.5%; Synovial sarcoma: 28.1%; Unclassified/undifferentiated sarcoma: 6.3%; Neurosarcoma: 21.9% |
| Ifosfamide | Scheulen 1983 | Phase II, SA | 16 | - | - | - | Anthracycline: 100% | - | STS (non-specified): 100% |
| Liposomal doxorubicin | Skubitz 2003 | Phase II, SA | 20 | - | 50% | - | Anthracycline and ifosfamide: 100% | - | Angiosarcoma: 5.0%; Fibrosarcoma: 5.0%; Leiomyosarcoma: 25.0%; Liposarcoma: 5.0%; MFH: 25.0%; Rhabdomyosarcoma: %; Synovial sarcoma: 5.0%; Alveolar soft part: 5.0%; Neurogenic: 10.0%; Extraosseus soft part: 5.0%; Hemangiopericytoma: 5.0%; Epithelioid: 5.0% |
| Liposomal doxorubicin | Toma 2000 | Phase II, SA | 25 | 60 (42 - 75) | - | - | Anthracycline: 100%; ifosfamide: 76.0% | - | Leiomyosarcoma: 32.0%; Liposarcoma: 20.0%; MFH: 28.0%; Synovial sarcoma: 8.0%; Neurofibrosarcoma: 4.0%; Clear cell sarcoma: 4.0%; Chondrosarcoma: 4.0% |
| Methotrexate | Buesa 1984 | Phase II, SA | 37 | 40^*^ (16 - 67) | 57.1% | - | Anthracycline: 100% | - | STS (non-specified): 100% |
| Paclitaxel | Palumbo 1997 | Phase II, SA | 12 | 63 (34 - 76) | 58% | - | Anthracycline/ifosfamide: 100% | PS 0, 1: 8 (66.7); PS 2: 4 (33.3) | Angiosarcoma: 8.3%; Leiomyosarcoma: 33.3%; MFH: 41.7%; Synovial sarcoma: 8.3%; Chondrosarcoma: 8.3% |
| Paclitaxel | Patel 1997 | Phase II, SA | 12 | - | - | - | Anthracycline and/or ifosfamide: 100% | - | Leiomyosarcoma: 8.3%; MFH: 8.3%; Rhabdomyosarcoma: 16.7%; Synovial sarcoma: 8.3%; STS (non-specified): 25.0%; Stromal sarcoma of breast: 25.0%; Alveolar soft part sarcoma: 8.3% |
| Paclitaxel | Skubitz 1997 | Phase I, SA | 17 | - | - | - | Anthracycline and ifosfamide: 100% | - | Angiosarcoma: 5.9%; Fibrosarcoma: 5.9%; Leiomyosarcoma: 58.8%; MFH: 11.8%; Synovial sarcoma: 11.8%; Cystosarcoma phalloides: 5.9% |
| Sorafenib | Bertuzzi 2010 | Phase II, SA | 61 | - | - | - | Anthracycline: 100% | - | Most frequent pathologic subtypes: leiomyosarcoma and liposarcoma |
| Sorafenib | Pacey 2011 | Phase II, SA | 16 | - | - | - | Anthracycline and/or ifosfamide: 100% | - | Fibrosarcoma: 12.5%; Leiomyosarcoma: 25.0%; Liposarcoma: 12.5%; Synovial sarcoma: 12.5%; Spindle cell: 25.0%; Clear cell sarcoma: 6.3%; Chondrosarcoma: 6.3% |
| Sunitinib | Decoster 2010 | Phase II, SA | 24 | - | - | - | Anthracycline: 100% | - | STS (non-specified): 100% |
| Trabectedin | Garcia-Carbonero 2004 | Phase II, SA | 36 | 48 (19 - 68) | 39% | 167.3 weeks (9.1 weeks - 191.1 weeks) | Anthracycline: 100%; ifosfamide: 83.3% | PS 0: 21 (58.3); PS 1: 15 (41.7) | Leiomyosarcoma: 36.1%; Liposarcoma: 27.8%; Synovial sarcoma: 16.7%; Malignant schwannoma: 5.6%; others: 13.9% |
| Trabectedin | Le Cesne 2005 | Phase II, SA | 104 | 53 (18 - 92) | 51% | 147.3 weeks | Anthracycline or ifosfamide: 100% | PS 0: 38^#^ (36.5); PS 1: 66^#^ (63.5) | Angiosarcoma: 1.0%; Fibrosarcoma: 1.0%; Leiomyosarcoma: 41.4%; Liposarcoma: 9.6%; MFH: 5.8%; Rhabdomyosarcoma: 1.0%; Synovial sarcoma: 17.3%; Miscellaneous: 8.7%; Undifferentiated: 6.7%; Unclassified:4.8%; Neurosarcoma 2.9% |
| Trabectedin | Yovine 2004 | Phase II, SA | 27 | - | - | 112.7 weeks (66.3 weeks - 168.6 weeks) | Anthracycline: 100% | - | Fibrosarcoma: 11.1%; Leiomyosarcoma: 48.2%; Liposarcoma: 3.7%; MFH: 3.7%; Synovial sarcoma: 7.4%; Others: 25.9% |
| Biricodar + doxorubicin | Bramwell 2002 | Phase I/II, SA | 18 | - | - | - | Anthracycline: 100% | - | Leiomyosarcoma: 50.0%; Liposarcoma: 5.6%; MFH: 5.6%; Synovial sarcoma: 11.1%; Spindle cell sarcoma: 11.1%; Malignant schwannoma: 5.6%; Malignant undifferentiated: 5.6%; Neurogenic sarcoma: 5.6% |
| Cisplatin + ifosfamide | Budd 1993 | Phase II, SA | 38 | (18 - 73) | 55.3% | - | Anthracycline: 97.4% | PS 0, 1: 30 (78.9)^†^;  PS 2: 8 (21.1) | Fibrosarcoma: 5.3%; Leiomyosarcoma: 50.0%; Liposarcoma: 5.3%; MFH: 5.3%; Rhabdomyosarcoma: 2.6%; Synovial sarcoma: 5.3%; Sarcoma, not otherwise specified: 13.2%; Extraskeletal chondrosarcoma: 5.3%; Extraskeletal osteosarcoma: 2.6%; Malignant peripheral-nerve sarcoma: 2.6%; Malignant granular-cell sarcoma: 2.6% |
| Cisplatin + vinblastine | Keohan 1997 | Phase II, SA | 18 | 52 (19 - 69) | 44.4% | - | Anthracycline: 100% | - | Leiomyosarcoma: 61.1%; MFH: 5.6%; Synovial sarcoma: 5.6%; Mesenchymal: 5.6%; Haemangiopericytoma: 5.6%; Myxoid: 5.6%; Neurofibrosarcoma: 5.6% |
| D + IL-2 | Le Cesne 1999 | Unclear, SA | 12 | - | - | - | Anthracycline: 100% | - | STS (non-specified): 100% |
| Epirubicin + lonidamine | Lopez 1995 | SA | 25 | 53 (21 - 73) | 56% | - | Anthracycline: 100% | - | Fibrosarcoma: 12.5%; Leiomyosarcoma: 29.2%; Liposarcoma: 8.3%; MFH: 16.7%; Hemangiopericytoma: 32.0%; all others: 24.0% |
| Etoposide + ifosfamide | Saeter 1995 | Unclear, SA | 11 | - | - | - | Anthracycline: 90.9% | - | STS (non-specified): 100% |
| Etoposide + ifosfamide | Skubitz 1993 | Phase I/II, SA | 16 | - | - | - | Anthracycline; 100% | - | Angiosarcoma: 6.3%; Fibrosarcoma: 6.3%; Leiomyosarcoma: 12.5%; Liposarcoma: 6.3%; MFH: 25.0%; Synovial sarcoma: 25.0%; Alveolar soft part sarcoma: 6.3%; Epithelioid sarcoma: 6.3%; Extraosseous osteosarcoma: 6.3% |
| Gemcitabine + dacarbazine | Buesa 2004 | Phase I, SA | 22 | 45.2 (29 - 77) | 36.4% | - | Anthracycline ; 18.2%; anthracycline and ifosfamide: 77.3%; other: 22.7^¶^ | PS 0: 13 (59.1); PS 1: 8 (36.4);  PS 2: 1 (4.6) | Leiomyosarcoma: 18.2%; Liposarcoma: 9.1%; MFH: 40.9%; Synovial sarcoma: 9.1%; Others: 22.7% |
| Gemcitabine + dacarbazine | Losa 2007 | Phase II, SA | 26 | 51 (24 - 75) | 65.4% | 145.0 weeks (114.0 weeks – 152.0 weeks) | Anthracycline: 92.3%; ifosfamide: 65.4%; anthracycline and ifosfamide: 65.4%; other: 7.7^^ | PS 0: 10 (38.5); PS 1: 12 (46.2); PS 2: 4 (15.4) | Leiomyosarcoma: 15.4%; Liposarcoma: 7.7%; MFH: 23.1%; Rhabdomyosarcoma: 11.5%; Neurosarcoma: 7.7%; Other or unclassified: 34.6% |
| Gemcitabine + docetaxel | Hensley 2002 | Phase II, SA | 16 | - | - | - | Anthracycline and/or ifosfamide: 100% | - | Leiomyosarcoma: 100% |
| Gemcitabine + docetaxel | Hensley 2008 | Phase II, SA | 51 | 50 (30 - 72) | 0% | - | Anthracycline; 89.6%; ifosfamide: 6.3%; other: 4.2^††^ | PS 0: 34^**^ (70.8); PS 1: 13^**^ (27.1); PS 2: 1^**^ (2.1) | Leiomyosarcoma: 100% |
| Gemcitabine + docetaxel | Montalar 2008 | Unclear, SA | 12 | 32 (18 - 56) | 83.3% | - | Anthracycline and ifosfamide: 100% | PS 0, 1: 9 (75.0); PS 2: 3 (25.0) | Fibrosarcoma: 8.3%; Liposarcoma: 25.0%; Synovial sarcoma: 50.0%; Gynaecologic sarcomas: 16.7% |
| Methotrexate + vincristine | Vaughn 1984 | Unclear, SA | 14 | - | - | - | Anthracycline: 100% | - | STS (non-specified): 100% |
| VAC + IE | Palumbo 1998 | Phase II, SA | 12 | - | - | - | Anthracycline or ifosfamide: 100% | - | STS (non-specified): 100% |
| C + VC + D + DTIC + IL-2 | Gravis 2001 | Phase II, n-RCT | 1 | 62^*^ | 0% | - | Anthracycline: 100% | - | Leiomyosarcoma: 100% |
| D + I + DTIC + IL-2 | Gravis 2001 | Phase II, n-RCT | 9 | 49 (38 - 58) | 44.4% | - | Anthracycline and ifosfamide: 100% | - | Leiomyosarcoma: 77.8%; Clear cell sarcoma: 11.1%; Chondrosarcoma: 11.1% |
| D + IL-2 | Gravis 2001 | Phase II, n-RCT | 3 | 48 (33 - 56) | 33.3% | - | Anthracycline and ifosfamide: 100% | - | Leiomyosarcoma: 33.3%; Liposarcoma: 33.3%; Synovial sarcoma: 33.3%; |
| Carboplatin + etoposide | Holstein 1996 | n-RCT | 8 | 42 (16.3)^$^ | 50% | - | Anthracycline and ifosfamide: 100% | - | Fibrosarcoma: 12.5%; Leiomyosarcoma: 50.0%; MFH: 12.5%; Rhabdomyosarcoma: 12.5%; Schwannoma grade III reported: 12.5% |
| Dacarbazine | Holstein 1996 | n-RCT | 14 | 52.1 (14.6)^$^ | 71.4% | - | Anthracycline and ifosfamide: 100% | - | Fibrosarcoma: 7.1%; Leiomyosarcoma: 35.7%; Liposarcoma: 7.1%; MFH: 7.1%; Rhabdomyosarcoma: 7.1%; Synovial sarcoma: 14.3%; Schwannoma: 14.3%; Undifferentiated sarcoma grade II: 7.1% |

C + VC + D + DTIC + IL-2: Cyclophosphamide + Vincristine + Doxorubicin + Dacarbazine + Interleukin-2; ECOG: Eastern Cooperative Oncology Group; D + IL-2: Doxorubicin + Interleukin-2; D + I + DTIC + IL-2: Doxorubicin + Ifosfamide + Dacarbazine + Interleukin-2; MPNST: Malignant Peripheral Nerve Sheath Tumour; N: Number of Included Patients; n: Number with Outcome; n-RCT: Non-Randomised Controlled Trials; PS: Performance Status; SA: Single Arm; VAC + IE: Vincristine + Adriamycin + Cyclophosphamide + Ifosfamide + Etoposide; ^*^Represents mean age; ^$^Represents mean (standard deviation) age; ^**^GOG performance status reported; ^#^Criteria unclear; †SCOG PS 0 to 1; ^Prior therapy with ifosfamide/cyclophosphamide; ‡Two patients received 5 mg/m2 ifosfamide + dacarbazine and one patient received 5 mg/m2 ifosfamide + doxorubicin-dacarbazine; ^¶^Two patients previously received ifosfamide + etoposide and three patients previously received other therapy; ^^Prior temozolomide therapy; ^††^Prior therapy with paclitaxel-cisplatin, temozolomide; -Represents data not reported.
