# Supplementary material for: Efficacy and safety of pharmacological interventions in second- or later-line treatment of patients with advanced soft tissue sarcoma: a systematic review
Source: BMC Cancer. 2013 Aug 13;13:385. doi: 10.1186/1471-2407-13-385 (PMC3765173; doi:10.1186/1471-2407-13-385)
Supplement: Additional file 6 — List of prospective small-size studies (sample size <10) and retrospective studies. This file describes the list of prospective small-size studies (sample size <10) and retrospective studies included in the review. [file 1471-2407-13-385-S6.doc]

Additional file 6 – List of prospective small-size studies (sample size <10) and retrospective studies

| **Study name** | **Title** | **Authors** |
| --- | --- | --- |
| Merimsky 2000 | Gemcitabine in soft tissue or bone sarcoma resistant to standard chemotherapy: A phase II study | Merimsky, O., Meller, I., Flusser, G., Kollender, Y., Issakov, J., Weil-Ben-Arush, M., Fenig, E., Neuman, G., Sapir, D., Ariad, S., Inbar, M. |
| Fata 1999 | Paclitaxel in the treatment of patients with anglosarcoma of the scalp or face | Fata, F., O`Reilly, E., Ilson, D., Pfister, D., Leffel, D., Kelsen, D.P., Schwartz, G.K., Casper, E.S. |
| Gian 1996 | A phase II trial of paclitaxel in the treatment of recurrent or metastatic soft tissue sarcomas or bone sarcomas. | Gian, V.G., Johnson, T.J., Marsh, R.W., Schuhmacher, C., Lynch, J.W. |
| Patel 1995 | Extraskeletal myxoid chondrosarcoma: Long-term experience with chemotherapy | Patel, S.R., Burgess, M.A., Papadopoulos, N.E., Linke, K.A., Benjamin, R.S. |
| Wiklund 1992 | Ifosfamide, vincristine, doxorubicin and dacarbazine in adult patients with advanced soft-tissue sarcoma | Wiklund, T.A., BLomqvist, C.P., Virolainen, M., Elomaa, I. |
| Lopez 1991 | Epirubicin and DTIC (EDIC) for advanced soft-tissue sarcomas | Lopez, M., Carpano, S., Di Lauro, L., Vici, P., Conti, E.M.S. |
| Mir 2011 | Feasibility of metronomic oral cyclophosphamide plus prednisolone in elderly patients with inoperable or metastatic soft tissue sarcoma | Mir, O., Domont, J., Cioffi, A., Bonvalot, S., Boulet, B., Le Pechoux, C., Terrier, P., Spielmann, M., Le Cesne, A. |
| Mir 2010 | Metronomic oral cyclophosphamide (CPM) and prednisolone in elderly patients (pts) with inoperable or metastatic soft tissue sarcoma (STS) | Mir, O., Domont, J., Cioffi, A., Bonvalot, S., Boulet, B., le Pechoux, C., Missenard, G., Rimareix, F., Terrier, P., Le Cesne, A. |
| Stacchiotti 2010 | Clear cell sarcoma (CCR): Clinical behavior and response to chemotherapy | Stacchiotti, S., Palassini, E., Negri, T., Orsenigo, M., Bertulli, R., Morosi, C., Pilotti, S., Fiore, M., Gronchi, A., Casali. P.G. |
| Italiano 2010 | Clinical outcome of leiomyosarcomas of vascular origin: Comparison with leiomyosarcomas of other origin | Italiano, A., Toulmonde, M., Stoeckle, E., Kind, M., Kantor, G., Coindre, J.-M., Bui, B. |
| Coriat 2010 | Ambulatory administration of 5-day infusion ifosfamide + mesna: A pilot study in sarcoma patients | Coriat, R., Mir, O., Camps, S., Ropert, S., Billemont, B., Leconte, M., Larousserie, F., Anract, P., Alexandre, J., Goldwasser, F. |
| Grenader 2009 | Long-term response to pegylated liposomal doxorubicin in patients with metastatic soft tissue sarcomas | Grenader, T., Goldberg, A., Hadas-Halperin, I., Gabizon, A. |
| Grenader 2008 | Long-term response to pegylated liposomal doxorubicin in patients with metastatic soft-tissue sarcomas | Grenader, T., Isacson, R., Segal, A., Gabizon, A. |
| George 2006 | Selective kinase inhibition with daily imatinib intensifies toxicity of chemotherapy in patients with solid tumours | George, S., Desai, J., Paul Eder, J., Manola, J., Ryan, D.P., Appleman, L.J., Demetri, G.D. |
| Skubitz 2005 | Paclitaxel and pegylated-liposomal doxorubicin are both active in angiosarcoma | Skubitz, K.M., Haddad, P.A. |
| Leu 2004 | Laboratory and clinical evidence of synergistic cytotoxicity of sequential treament with gemcitabine followed by docetaxel in the treatment of sarcoma | Leu, K.M., Ostruszka, L.J., Shewach, D., Zalupski, M., Sondak, V., Sybil Biertnann, J., Lee, J.S.-J., Couwlier, C., Palazzolo, K., Baker, L.H. |
| Yalcin 2004 | High-dose ifosfamide with hematopoietic growth factor support in advanced bone and soft tissue sarcomas | Yalcin, B., Pamir, A., Buyukcelik, A., Utkan, G., Akbulut, H., Demirkazik, A., Icli, F. |
| Fiegl 2004 | Ifosfamide, carboplatin and etoposide (ICE) as second-line regimen alone and in combination with regional hyperthermia is active in chemo-pre-treated advanced soft tissue sarcoma of adults | Fiegl, M., Schlemmer, M., Wendtner, C.-M., Abdel-Rahman, S., Fahn, W., Issels, R.D. |
| Chow 2004 | Feasibility and pharmacokinetic study of infusional dexrazoxane and dose-intensive doxorubicin administered concurrently over 96 h for the treatment of advanced malignancies | Chow, W.A., Synold, T.W., Tetef, M.L., Longmate, J., Frankel, P., Lawrence, J., Al-Khadimi, Z., Leong, L., Lim, D., Margolin, K., Morgan Jr., R.J., Raschko, J., Shibata, S., Somlo, G., Twardowski, P., Yen, Y., Doroshow, J.H. |
| Cartei 2003 | Dose finding of ifosfamide administered with a chronic two-week continuous infusion | Cartei, G., Clocchiatti, L., Sacco, C., Pella, N., Bearz, A., Mantero, J., Pastorelli, D., Salmaso, F., Zustovich, F. |
| Patel 2001 | Phase II clinical investigation of gemcitabine in advanced soft tissue sarcomas and window evaluation of dose rate on gemcitabine triphosphate accumulation | Patel, S.R., Gandhi, V., Jenkins, J., Papadopolous, N., Burgess, M.A., Plager, C., Plunkett, W., Benjamin, R.S. |
| Jones 2009 | Palliative Chemotherapy in Epithelioid Sarcoma | Jones, R.L., Constantinidou, A.,Thway, K., Fisher, C., Al-Muderis, O., Scurr, M., Judson, I.R. |
| Stacchiotti 2008 | Response to sunitinib malate (SM) in alveolar soft part sarcoma (ASPS) | Stacchiotti, S., Tamborini, E., Bertulli, R., Piovesan, C., Marrari, A., Morosi, C., Crippa, F., Pilotti, S., Gronchi, A., Casali, P.G. |
| Rahal 2010 | Trabectedin: Still active beyond progression in advanced soft tissue sarcomas | Rahal, A., Benbrahim, Z., Glaoui, M., Cioffi, A., Domont, J., Terrier, P., Bonvalot, S., Le Pechoux, C., Boulet, B., Le Cesne, A. |
| Hare 2011 | Phase II study of biweekly pemetrexed and gemcitabine in patients with previously treated advanced soft tissue sarcoma | Hare,E.G.; Visser,C.M.; Matushansky,I.; Taub,R.N. |
| Italiano 2011 | Temsirolimus in advanced leiomyosarcomas: Patterns of response and correlation with the activation of the mammalian target of rapamycin pathway | Temsirolimus in advanced leiomyosarcomas: Patterns of response and correlation with the activation of the mammalian target of rapamycin pathway |
