# Supplementary material for: Efficacy and safety of pharmacological interventions in second- or later-line treatment of patients with advanced soft tissue sarcoma: a systematic review
Source: BMC Cancer. 2013 Aug 13;13:385. doi: 10.1186/1471-2407-13-385 (PMC3765173; doi:10.1186/1471-2407-13-385)
Supplement: Additional file 7 — Summary of grade 3 or grade 4 specific adverse events observed across non-randomised trials. This file describes the grade 3/4 AEs observed across non-randomised controlled trials. [file 1471-2407-13-385-S7.doc]

Additional file 7 – Summary of grade 3 or grade 4 specific adverse events observed across non-randomised trials

| Treatment | Study | Anaemia, n (%) | Leucopenia, n (%) | Neutropenia, n (%) | Thrombocytopenia, n (%) | Diarrhoea, n (%) | Mucositis, n (%) | Nausea, n (%) | Nausea and vomiting, n (%) | Stomatitis, n (%) | Vomiting, n (%) | Fatigue, n (%) | Fever, n (%) | Allergy/Hypersensitivity, n (%) | ALT increase, n (%) | ALP increase, n (%) | AST increase, n (%) | Bilirubin increase, n (%) | CNS/neurotoxicity/  cerebral toxicity, n (%) | Alopecia, n (%) | Infection, n (%) |
| --- | --- | --- | --- | --- | --- | --- | --- | --- | --- | --- | --- | --- | --- | --- | --- | --- | --- | --- | --- | --- | --- |
| Cisplatin | Thigpen 1986 | - | - | - | - | - | - | - | 2  (10.5) | - | - | - | - | - | - | - | - | - | - | - | - |
| Cyclophosphamide | Bramwell 1993 | - | - | - | - | 0 (0.0) ^$^ | - | - | 0 (0.0)^$^ | - | - | - | - | - | - | - | - | - | 0 (0.0) | - | 0 (0.0)^$^ |
| Dacarbazine | Buesa 1991 | 6 (13.6) | 16 (36.4) | - | 12 (27.3) | 2 (4.5) | - | - | 18 (40.9) | - | - | - | - | - | - | - | - | - | - | - | - |
| Docetaxel | Kostler 2001 | - | - | - | 0 (0.0) | - | - | 0 (0.0) | - | - | - | - | - | - | - | - | - | - | - | - | - |
| Docetaxel | Santoro 1999 | 3 (8.3) | 28 (77.8) | 32 (88.9) | 1 (2.8) | 2 (5.6) | 3 (8.3) | - | - | - | 1 (2.8) | - | 1 (2.8) | 2 (5.6) | - | - | - | - | 0 (0.0) | - | 5 (13.9) |
| Docetaxel | Van Hoesel 1994 | 0 (0.0)^$^ | - | - | 0 (0.0) | 0 (0.0) | - | 0 (0.0) ^$^ | - | - | 0 (0.0)~ | - | - | - | - | - | - | - | - | - | - |
| Etoposide | Crawley 1997 | - | - | 13 (76.5)^$^ | 1 (5.9)^$^ | - | - | - | - | - | 2 (11.8)~ | - | - | - | - | - | - | - | - | - | - |
| Etoposide | Dombern-owsky 1987 | - | - | - | 4 (12.9)~ | - | - | - | - | - | - | - | - | - | - | - | - | - | - | - | - |
| Gefitinib | Ray-Coquard 2008 | - | - | - | - | 1 (2.1) | - | 0 (0.0) | - | 0 (0.0) | 0 (0.0) | 5 (10.4) | 0 (0.0) | - | 1 (2.1) | 0 (0.0) | 0 (0.0) | 1 (2.1) | - | 0 (0.0) | - |
| Gemcitabine | Ferraresi 2008 | 0 (0.0) | 2 (14.3) | 2 (14.3) | 1 (7.1) | - | 0 (0.0) | - | 0 (0.0) | - | - | - | - | - | - | - | - | 0 (0.0) | - | - | - |
| Gemcitabine | Hartmann 2006 | 1 (6.7) | - | 2 (13.3) | 5 (33.3) | 0 (0.0) | - | - | 0 (0.0) | - | - | - | 0 (0.0) | - | 0 (0.0) | - | 0 (0.0) | - | - | 1 (6.7) | - |
| Gemcitabine | Look 2004 | 0 (0.0)^$^ | 8 (24.2) | 11 (33.3) | 3 (9.1) | - | - | - | - | - | - | 0 (0.0) | 0 (0.0) | - | - | 0 (0.0) | 0 (0.0)^$^ | - | 0 (0.0)^$^ | - | - |
| Gemcitabine | Spath-Schwalbe 2000 | 0 (0.0) | - | 5 (27.8) | 1 (5.6) | - | 0 (0.0) | - | 0 (0.0) | - | - | - | 0 (0.0) | - | - | - | - | - | - | - | - |
| Ifosfamide | Le Cesne 1995 | 10 (25.0) | - | 40 (100.0) | 8 (20.0) | 0 (0.0) | - | - | - | - | - | - | - | - | - | - | - | - | 7 (17.5)~ | - | - |
| Ifosfamide | Nielson 2000 | - | - | - | - | - | - | 0 (0.0)^$^ | - | - | - | - | - | - | - | - | - | - | - | 0 (0.0)^$^ | - |
| Ifosfamide | Palumbo 1997a | - | - | 6 (15.8)^$^ | 7 (18.4) | - | - | - | - | - | - | - | - | - | - | - | - | - | - | - | - |
| Liposomal Doxorubicin | Toma 2000 | 0 (0.0)~ | - | - | 0 (0.0)~ | - | 0 (0.0)~ | - | 0 (0.0)~ | - | - | - | - | 0 (0.0)~ | - | - | - | - | - | 12 (48.0)~ | - |
| Methotrexate | Buesa 1984 | - | 6 (20.7)~ | - | 4 (13.8)~ | - | - | - | - | 8 (27.6)^#~^ | - | - | - | - | - | - | - | - | - | - | - |
| Paclitaxel | Skubitz 1997 | - | - | - | 0 (0.0) | 0 (0.0) | 0 (0.0) | 0 (0.0) | - | - | - | - | - | - | - | - | - | - | - | - | - |
| Paclitaxel | Palumbo 1997b | 1 (8.3) ~ | 0 (0.0)~ | 2 (16.7)~ | 0 (0.0)~ | - | - | - | 0 (0.0)~ | 0 (0.0)~ | - | - | 0 (0.0)~ | - | - | - | - | - | - | 0 (0.0)~ | 0 (0.0)~ |
| Sorafenib | Pacey 2011 | - | - | - | - | - | - | - | - | - | - | - | - | - | 0 (0.0) | - | 0 (0.0) | 0 (0.0) | - | 0 (0.0) | - |
| Sorafenib | Bertuzzi 2010 | - | - | - | - | 4 (6.6) |  | - | - | - | - | 5 (8.2) | - | - | - | - | - | - |  |  | - |
| Trabectedin | Garcia-Carbonero 2004 | 3 (8.3) | 15 (41.7) | 12 (33.3) | 6 (16.7) | - | - | 2 (5.6) | - | - | 1 (2.8) | 0 (0.0) | - | - | 7 (19.4) | 0 (0.0) | 9 (25.0) | 1 (2.8) | - | - | - |
| Trabectedin | Le Cesne 2005 | 16 (16.2)^*^ | 41 (41.4)^*^ | 52 (52.5)^*^ | 18 (18.2)^*^ | - | - | 7 (7.1) | - | - | 9 (9.1) | - | - | - | 44 (44.4) | - | 35 (35.4) | - | - | - | - |
| Trabectedin | Yovine 2004 | - | - | - | - | 0 (0.0) | - | - | - | 0 (0.0) | - |  | - | 0 (0.0) | - | - | - | 0 (0.0) | - | 0 (0.0) | 0 (0.0) |
| Cisplatin + ifosfamide | Budd 1993 | 9 (23.7) | 31 (81.6) | - | 11 (28.9) | - | - | - | - | - | - | - | - | - | - | - | - | - | 3 (7.9) | - | - |
| Gemcitabine + dacarbazine | Losa 2007 | 6 (23.1) | 12 (46.2) | - | 3 (11.5) | 0 (0.0)~ | - | 0 (0.0)~ | - | 0 (0.0)~ | 1 (3.8)~ | - | 0 (0.0)~ | - | 5 (19.2)~ | 0 (0.0)~ | 5 (19.2)~ | 0 (0.0)~ | - | 0 (0.0)~ | - |
| Gemcitabine + docetaxel | Hensley 2002 | - | - | - | - | - | - | - | - | - | - | 0 (0.0)^$^ | - | 0 (0.0)^$^ | - | - | - | - | - | - | - |
| Gemcitabine + docetaxel | Hensley 2008 | 12 (25.0) | 11 (22.9) | 10 (20.8) | 19  (39.6) | - | - | - | 1 (2.1) | - | - | 1 (2.1) | 2 (4.2) | 0 (0.0) | - | 0 (0.0) | 0 (0.0) | - | 0 (0.0) | 0 (0.0) | 3 (6.3) |
| Gemcitabine + docetaxel | Montalar 2008 | 4 (33.3) | - | 10 (83.3) | 2 (16.7) | - | - | - | - | - | - | - | - | - |  | - | - | - | - | - | - |
| Epirubicin + lonidamine | Lopez 1995 | - | 3 (12.0) | - | 2 (8.0) | - | - | - | 2 (8.0)~ | 1 (4.0)~ | - | - | - | - | - | - | - | - | - | - | - |
| Etoposide + ifosfamide | Skubitz 1993 | - | - | - | - | 0 (0.0) | - | 0 (0.0)~ | - | - | 0 (0.0) | - | - | - | - | - | - | - | - | - | - |
| VAC + IE | Palumbo 1998 | 0 (0.0)^$^ | - | - | 0 (0.0) | - | - | 0 (0.0) | - | - |  | - | - | - | - | - | - | - | - | - | - |
| Dacarbazine | Holstein 1996 | 0 (0.0) | 2* (14.3) | - | 0 (0.0)* | - | - | - | - | - | - | - | - | - | - | - | - | - | - | - | - |
| Carboplatin + etoposide | Holstein 1996 | 2 (25.0) | 6* (75.0) | - | 7 (87.5) | - | - | - | - | - | - | - | - | - | - | - | - | - | - | - | - |
| D + I + DTIC + IL-2 | Gravis 2001 | 1 (11.1) | - | - | - | - | - | - | - | - | - | - | - | - | - | - | - | - | - | - | - |

ALP: Alkaline Phosphatase; ALT: Alanine Aminotransferase; AST: Aspartate Aminotransferase; CNS: Central Nervous System; n: Number with Outcome; VAC + IE: Vincristine + Adriamycin + Cyclophosphamide + Ifosfamide + Etoposide; ^#^Severe stomatitis reported; -Represents data not reported; *Reported as toxicity on haemoglobin, leukocytes, granulocytes, lymphocytes, and platelet; ~Grade 3 Reported; $Grade 4 reported.
